# Response of a General Restricted Open-Shell Hartree–Fock Wave Function. I: Formalism, Analytic Gradients, and Electric and Magnetic Response Properties

**DOI:** 10.1021/acs.jpca.5c05207

**Published:** 2025-10-10

**Authors:** Frank Neese

**Affiliations:** Department of Molecular Theory and Spectroscopy, 28314 Max Planck Institut für Kohlenforschung, Kaiser Wilhelm Platz 1, Mülheim an der Ruhr D-45470, Germany

## Abstract

In this work, the formal development and implementation of a general restricted open-shell Hartree–Fock (g-ROHF) response theory is presented. The theory enables analytic computation of electric and magnetic response properties for arbitrarily complex open-shell configurations. In contrast to traditional ROHF methods, which are typically restricted to high-spin cases, the g-ROHF formulation supports general-spin couplings and orbital degeneracies while preserving the spin purity. A new set of vector-coupling coefficients is introduced that allows for the calculation of a proper spin density from a g-ROHF wave function. Analytic nuclear derivatives, along with the electric and magnetic orbital Hessians, are derived in a unified framework. Special attention is given to the treatment of SCF instabilities and the projection of unphysical modes from the response space. An efficient AO-driven implementation is described and validated across a broad range of open-shell systems, including small molecules, transition-metal complexes, and metal–radical assemblies. Specifically, the method is applied to the calculation of g-tensors and hyperfine couplings (including spin–orbit coupling corrections) in experimentally well-characterized systems such as mixed-valence manganese­(III/IV) dimers and the metal–radical complex Fe­(GMA)­(pyridine)^+^. The g-ROHF framework provides a robust, efficient, and physically rigorous platform for treating the electronic structure and properties of complex open-shell molecules and serves as a convenient foundation for the development of post-Hartree–Fock correlation methods. The present work sets the stage for extensions to excited-state response theory, DFT-based treatments, and coupled-cluster response formulations.

## Graphical abstract

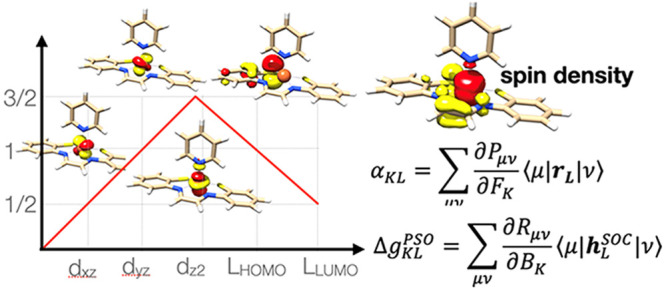

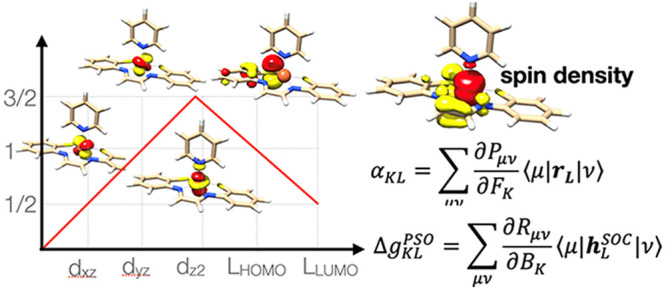

## 1 Introduction

The quantum mechanics of electron spin is one of the most fascinating subjects one faces in the application of quantum mechanics in chemistry, probably because it has no counterpart in classical physics. Due to their spin, electrons behave as peculiar bar magnets that can adopt only discrete orientations relative to a chosen quantization axis. This leads to rather complex and puzzling behavior, in particular, when several unpaired electrons couple to a resulting total spin. Early on in the development of quantum mechanics, a great level of attention was given to this subject, in particular in the framework of the interpretation of the complex multiplet structure in the spectra of free atoms and ions.^1,2^

Of course, the electron spin underlies many molecular magnetic phenomena, ranging from magnetic susceptibility and EPR spectroscopy to the design of single-molecule magnets for quantum technologies.^3,4^ Fortunately, it turned out that many of the intricate challenges of dealing with the electron spin can be absorbed into an effective spin-Hamiltonian that contains only spin-operators and external field variables^5,6^ In this way, the complexities arising from the coupling of several unpaired electrons to a given spin multiplet can be absorbed into the effective spin-Hamiltonian parameters. This allows researchers to work with the effective manifold of only 2S + 1 magnetic sublevels |*SM*⟩ where *M* = 1, *S* – 1, ..., and −*S*. While dealing with the spin-Hamiltonian instead of the full, possibly relativistic, molecular Hamiltonian represents an enormous simplification, the important subject of when the construction is even valid has become somewhat neglected over time.^4^

In making the connection between the spin-Hamiltonian and the molecular Hamiltonian, various pathways can be taken. In modern approaches, the route proceeding through analytic derivative theory^7–11^ has arguably been the most successful since it leads to elegant and efficient as well as unambiguously defined working equations.^12,13^ In particular, it is important that all 2S + 1 members of the given spin Multiplet |Ψ^*SM*^⟩ share the same spatial part of the wave function and differ only in their spin parts. This allows one to apply the powerful Wigner–Eckart theorem to reduce the problem to only the calculation of matrix elements of the principal component (M=S) of a given multiplet |Ψ^*SM*^⟩.^14^

Thus, the quantum chemical problem at hand is to find an effective means by which such a principal component |Ψ^*SS*^⟩ can be constructed. To set the stage, let us briefly revisit the development of open-shell methods in quantum chemistry.

It is noteworthy that the earliest formulations of self-consistent field (SCF) theory by Roothaan^15,16^ already exploited a rather general formalism for open-shell situations that was able to cover many of the atomic multiplets that were met in practice.^17^ Application of this formalism required practitioners to derive a set of vector-coupling coefficients that are specific to a given open-shell case. Since this posed a significant barrier, this art was slowly forgotten, and instead the spin-unrestricted Hartree–Fock (UHF) method of Pople and Nesbet gained popularity.^18^ In this method, one only differentiates between spin-up and spin-down electrons and assigns different orbitals to each “spin-channel”. While this leads to a straightforward black box methodology, the spin-unrestricted method is somewhat problematic. First of all, it always suffers from spin-contamination and, in general, is unable to retain the total spin S as a good quantum number. The effects of spin-contamination (an undesired effect) and spin-polarization (a desired effect) then become difficult to differentiate. However, more importantly, more complex spin–coupling situations such as those that occur in orbitally degenerate systems or in antiferromagnetically coupled systems, cannot be described properly with the spin-unrestricted methodology.

The counterpart of the UHF method is usually referred to as a restricted open-shell Hartree–Fock (ROHF) method.^15,16,19^ It is very often used exclusively in the context of high-spin open-shell situations in which n-unpaired electrons occupy n-orbitals to couple to a total spin of *S* = *n*/2. This high-spin case is the only open-shell situation that UHF describes approximately correctly. It turns out that ROHF applied to a high-spin open-shell situation offers, at best, moderate advantages over UHF. It is a spin-eigenfunction and, if implemented correctly, is also computationally somewhat cheaper than UHF. As shown, for example, by Tsuchimochi and Scuseria,^20^ ROHF for a high-spin case can be implemented efficiently using essentially UHF machinery. ROHF, however, often suffers from poorer convergence than UHF and the ROHF energy is always higher than the UHF energy, as expected from its limited variational freedom. As a result, ROHF is not frequently used in computational chemistry studies despite the fact that it often represents a much better starting point for correlated calculations, e.g., coupled-cluster studies, than UHF treatments.^21–23^

When it comes to more complex spin–coupling situations, the current default way is to resort to multiconfigurational methods, such as CASSCF.^24^ If all open-shell orbitals are part of the active space, then the CASSCF method, by construction, captures all the intricacies of spin–coupling. While CASSCF is very powerful, it is also a somewhat indiscriminate and expensive way to deal with the spin–coupling problem. It is well-known that the computational complexity of CASSCF increases roughly factorially with the size of the active space.^25^ Thus, there is a lot of computation involved in order to determine coefficients that are fixed by spin symmetry.^19^ Since CASSCF contains all physical effects inside the active space, it is an all-or-nothing approach, and one also loses the ability to take a more fine-grained approach to the open-shell problem at hand. In fact, it becomes difficult to isolate specific spin–coupling situations and their impact on the overall electronic structure and properties. While impressive progress has been made in the development and application of approximate large-scale CASSCF methods.^26,27^ it is still desirable to look for economic electronic structure methods that do as little work as possible and as much work as necessary.

One method that can be thought of as following this spirit is the spin-flip methodology proposed and extensively developed by Krylov, Head-Gordon and co-workers.^28–30^ (for a recent review see^31^). In this approach, one starts from a single determinant of the high-spin type in which all unpaired electrons are aligned in parallel. Subsequently, spin-flip operators are applied to access lower multiplicities and more complex spin–coupling situations. Spin-flip can be combined with a host of different electronic structure methods and provides an elegant and quite successful approach to more complex open-shell spin–coupling situations.^31^

In this paper, we take an alternative approach and explore general ROHF (g-ROHF) theory as a compelling middle ground between the structural shortcomings of UHF, and the computational demands of CASSCF. While one might view this as an extension of the well-known and widely available ROHF theory for high-spin states, the formalism, in its essence, has been around in the community since the pioneering work of Clemens Roothaan.^15,16^ A number of authors have subsequently contributed to general ROHF theory.^19,32–37^ An important contribution is the work by Edwards and Zerner that clearly laid out the computational procedure and showed its application to the multiplets arising from open-shell transition-metal ions^19^ and even systems as complex as the active site of nitrogenase.^38,39^ Zerner and co-workers then formulated highly useful ROHF variants that are able to capture the average of a set of configuration (configuration-averaged HF, CAHF^40^) or over all states of a given spin (spin-averaged HF, SAHF^41^) that often can substitute for CASSCF at greatly reduced computational cost. The paper by Edwards and Zerner makes explicit and detailed reference to earlier work by Huzinaga^17^ and Hirao and Nakatsuji.^42^ An equivalent mathematical formulation was described in the excellent monograph of Carbo and Riera^43^ and the associated paper by Caballol et al.^44^ that was also taken up in the work by Fernandez-Rico and co-workers on SCF convergence acceleration.^34,35^

While all of the mentioned authors have focused on the solution of the SCF equation and the ROHF energy, rather limited attention has been given to the calculation of general ROHF response properties. It surely should be noted that high-spin ROHF response treatments have been quite common and have been developed to a very high sophistication in the DALTON program.^45–48^ High-spin ROHF response properties and Hessians are also available in the DALTON,^49^ GAMESS,^50^ and NWChem^51^ programs.

The purpose of this paper is to present a derivation and implementation of the response of the g-ROHF method. While the general ROHF method has been available in the ORCA package since its earliest beginnings, it has been relatively underutilized. A relatively early focus has been the construction of the ROCIS methodology that constructs a set of three spin-adapted CI-singles problems of total spin *S*, *S*–1, and *S*+1 based on a high-spin ROHF reference state with spin S.^37,52,53^ The roots of these three sets of CI solutions can then interact via quasi-degenerate perturbation theory (QDPT) through spin–orbit coupling (SOC) and external fields. This methodology has found widespread application in the field of X-ray absorption spectroscopy, given its very favorable cost-to-performance ratio, in particular, following the extension to the pair-natural orbital (PNO)-ROCIS variant that can be applied to very large molecules. More recently, our interest in general ROHF methods has been renewed with the development of the configuration state function (CSF)-ROHF method, a method that automatically constructs and converges the appropriate ROHF wave function for an arbitrarily complex CSF.^54^ Clearly, CSF-ROHF is a special case of g-ROHF given its restriction to a single spatial configuration. In follow-up papers, we demonstrated how to build a CI expansion on top of a CSF-ROHF ground state wave function, thus defining the General-Spin (GS)-ROCIS method^55^ that can be applied to systems as complex as antiferromagnetically coupled solids. The latest development is the extension of GS-ROCIS to cover SOC effects, which leads to a method that can be successfully applied, for example, to magnetic-circular dichroism (MCD) spectra and other situations.^56^

Given the usefulness of g-ROHF it is surprising that only limited formal development has been done on this very elegant formalism. This paper aims to fill this gap by formulating the necessary g-ROHF theory for the calculation of electric and magnetic response properties. We also devote some attention to the treatment of instabilities in the SCF solutions. Subsequent publications will deal with the calculation of excited states, analytic nuclear Hessians, and other extensions.

## 2 Theory

### 2.1 The ROHF Energy

We utilize the standard clamped-nuclei Born–Oppenheimer Hamiltonian:

1 $$H=V_{\mathrm{NN}}+\sum_{\mathrm{pq}}E_{p}^{q}h_{\mathrm{pq}}+\frac{1}{2}\sum_{\mathrm{pqrs}}\left(\mathrm{pq}|\mathrm{rs}\right)\left\{E_{p}^{q}E_{r}^{s}-\delta_{\mathrm{qr}}E_{p}^{s}\right\}$$

Where *V*_*NN*_ is the nuclear repulsion energy, *E*_*p*_^*q*^ = a_*p*α_^+^ *a*_*q*α_ + *a*_*p*β_^+^ *a*_*q*β_ is the familiar spin-traced orbital replacement operator in second quantization referring to an orthonormal orbital basis {*q*}, *h_pq_* is a matrix element of the one-electron operator, and (*pq* | *rs*) is a two-electron integral in Mulliken notation. Let Ψ be a many-electron wave function of the general ROHF type. Some examples of such wave functions are given in Table 1, where (*c*) represents a set (“core”) of doubly occupied orbitals and s_1_... s_*N*_ denote open-shell orbitals. A Slater determinant is denoted |*ijklmn*...|. An overbar indicates occupation with a spin-down electron and a walk through the branching diagram that defines a spin-eigenfunction is indicated by “+” if the electron in that position is coupled parallel to the total spin and “–” if it is coupled antiparallel to the total spin.

**Table 1:** Wave functions for Some Open-Shell Cases That Can Be Handled by g-ROHF

case	spin	branching diagram	wave function
high-spin	$S=\frac{1}{2}N$	+ + ... +	|Ψ = |(c)s_1_...s_*N*_|
open-shell singlet	*S* = 0	+ –	$|\Psi \rangle =\frac{1}{\sqrt{2}}\left(\left|\left(c\right)s_{1}{\mathrm{s̅}}_{2}\right|-\left|\left(c\right){\mathrm{s̅}}_{1}s_{2}\right|\right)$
doubly degenerate doublet	$S=\frac{1}{2}$	+0/0 +	$|\Psi \rangle =\frac{1}{\sqrt{2}}\left(\left|\left(c\right)s_{1}\right|+\left|\left(c\right)s_{2}\right|\right)$
sing-doublet	$S=\frac{1}{2}$	+ + –	$|\Psi \rangle =\frac{1}{\sqrt{2}}\left(-\left|\left(c\right){\mathrm{s̅}}_{1}s_{2}s_{3}\right|+\left|\left(c\right)s_{1}{\mathrm{s̅}}_{2}s_{3}\right|\right)$
trip-doublet	$S=\frac{1}{2}$	+ – +	$|\Psi \rangle =\frac{1}{\sqrt{6}}\left(2\left|\left(c\right)s_{1}s_{2}{\mathrm{s̅}}_{3}\right|-\left|\left(c\right){\mathrm{s̅}}_{1}s_{2}s_{3}\right|-\left|\left(c\right)s_{1}{\mathrm{s̅}}_{2}s_{3}\right|\right)$
antiferromagnetic triplets	*S* = 0	+ + – –	$|\Psi \rangle =\frac{1}{2\sqrt{3}}\left(2\left|\left(c\right)s_{1}s_{2}{\mathrm{s̅}}_{3}{\mathrm{s̅}}_{4}\right|-\left|\left(c\right){\mathrm{s̅}}_{1}s_{2}s_{3}{\mathrm{s̅}}_{4}\right|-\left|\left(c\right){\mathrm{s̅}}_{1}s_{2}{\mathrm{s̅}}_{3}s_{4}\right|-\left|\left(c\right)s_{1}{\mathrm{s̅}}_{2}s_{3}{\mathrm{s̅}}_{4}\right|-\left|\left(c\right)s_{1}{\mathrm{s̅}}_{2}{\mathrm{s̅}}_{3}s_{4}\right|+2\left|\left(c\right){\mathrm{s̅}}_{1}{\mathrm{s̅}}_{2}s_{3}s_{4}\right|\right)$
two open-singlets	*S* = 0	+ – + –	$|\Psi \rangle =\frac{1}{2}\left(-\left|\left(c\right){\mathrm{s̅}}_{1}s_{2}s_{3}{\mathrm{s̅}}_{4}\right|+\left|\left(c\right){\mathrm{s̅}}_{1}s_{2}{\mathrm{s̅}}_{3}s_{4}\right|+\left|\left(c\right)s_{1}{\mathrm{s̅}}_{2}s_{3}{\mathrm{s̅}}_{4}\right|-\left|\left(c\right)s_{1}{\mathrm{s̅}}_{2}{\mathrm{s̅}}_{3}s_{4}\right|\right)$
quartet-radical antiferromagnetic	*S* = 1	+ + + –	$|\Psi \rangle =\frac{\sqrt{3}}{6}\left(3\left|\left(c\right)s_{1}s_{2}s_{3}{\mathrm{s̅}}_{4}\right|-\left|\left(c\right){\mathrm{s̅}}_{1}s_{2}s_{3}s_{4}\right|-\left|\left(c\right)s_{1}{\mathrm{s̅}}_{2}s_{3}s_{4}\right|-\left|\left(c\right)s_{1}s_{2}{\mathrm{s̅}}_{3}s_{4}\right|\right)$

It should be noted that the wave functions in Table 1 are not invariant with respect to a unitary transformation among the partially occupied orbitals. Hence, care is required to obtain the desired orbital ordering. This will perhaps require a dedicated initial guess that may also involve orbital localization.

With the exception of the CAHF and related treatments, such wave functions are usually constructed to be eigenfunctions of the total spin and hence the total spin *S* is a good quantum number. The common feature of all of these electronic situations is that the wave function energy, expressed as the expectation value of the Hamiltonian, can be expressed in a form where only three types of integrals occur: (1) one-electron integrals *h*_*pp*_, (2) two-electron integrals of the Coulomb type (*pp*|*qq*), and (3) two-electron integrals of the exchange type, (*pq*|*pq*). Furthermore, the orbitals are not spin-dependent, but there is a single set of orbitals that describes the entire system. Finally, the orbitals can be grouped into an arbitrary number of shells *I* = 0.. *N*_*S*_ – 1 with *M*_*I*_ being the number of orbitals in shell *I* and *N*_*I*_ being the number of electrons in shell *I*. The first shell “0” contains the closed-shell, and subsequent shells are partially filled open shells.

Thus, in g-ROHF theory, the energy can be expressed as

2 $$\left\langle \Psi \left|H\right|\Psi \right\rangle =E^{\left(\mathrm{ROHF}\right)}=V_{\mathrm{NN}}+\sum_{I}\sum_{i_{I}\;}n_{I}h_{i_{I}i_{I}}+\frac{1}{4}\sum_{I,J}\sum_{i_{I}\;,j_{J}\;}n_{I}n_{J}\left\{2a^{\mathrm{IJ}}\left(i_{I}i_{I}|j_{J}j_{J}\right)-b^{\mathrm{IJ}}\left(i_{I}j_{J}|i_{I}j_{J}\right)\right\}$$

Here, *p*_*I*_ is an orbital that is a member of shell *I*, *n*_*I*_ is the average occupation number of an orbital in shell *I* obtained from $n_{I}=\frac{N_{I}}{M_{I}}$ . The vector-coupling coefficients *a* ^*IJ*^ and *b* ^*IJ*^ depend on the specific form of the underlying wave function and are obtained by writing out ⟨Ψ|*H*|Ψ⟩ and comparing it with the above energy expression in eq 2. The specific form of eq 2 is consistent with the paper by Edwards and Zerner. Obviously, the Edwards–Zerner equation is engineered to make it look as similar to the closed-shell case as possible. There is an alternative version due to Carbo and Riera, as also used by Fernandez–Rico and co-workers, that is aimed at avoiding all awkward factors of 2 or 1/2. It reads:

3 $$\left\langle \Psi \left|H\right|\Psi \right\rangle =E^{\left(\mathrm{ROHF}\right)}=V_{\mathrm{NN}}+\sum_{I}\sum_{i_{I}}\omega_{I}h_{i_{I}i_{I}}+\sum_{I,J}\sum_{i_{I}\;,j_{J}\;}\left\{\alpha_{\mathrm{IJ}}\left(i_{I}i_{I}|j_{J}j_{J}\right)-\beta_{\mathrm{IJ}}\left(i_{I}j_{J}|i_{I}j_{J}\right)\right\}$$

The connection between the formalisms is readily established to be

4 $$\begin{array}{l} \omega_{I}=n_{I}\\ \alpha_{\mathrm{IJ}}=\frac{1}{2}\omega_{I}\omega_{J}a_{\mathrm{IJ}}\\ \beta_{\mathrm{IJ}}=\frac{1}{4}\omega_{I}\omega_{J}b_{\mathrm{IJ}} \end{array}$$

This form is used for the remainder of the paper. Below a fourth set of vector-coupling coefficients, γ_*I*_, will be introduced, which will serve to describe the contributions of shell *I* to the spin density.

The relevant vector-coupling coefficients are listed in Figure 1.

**Figure 1:**
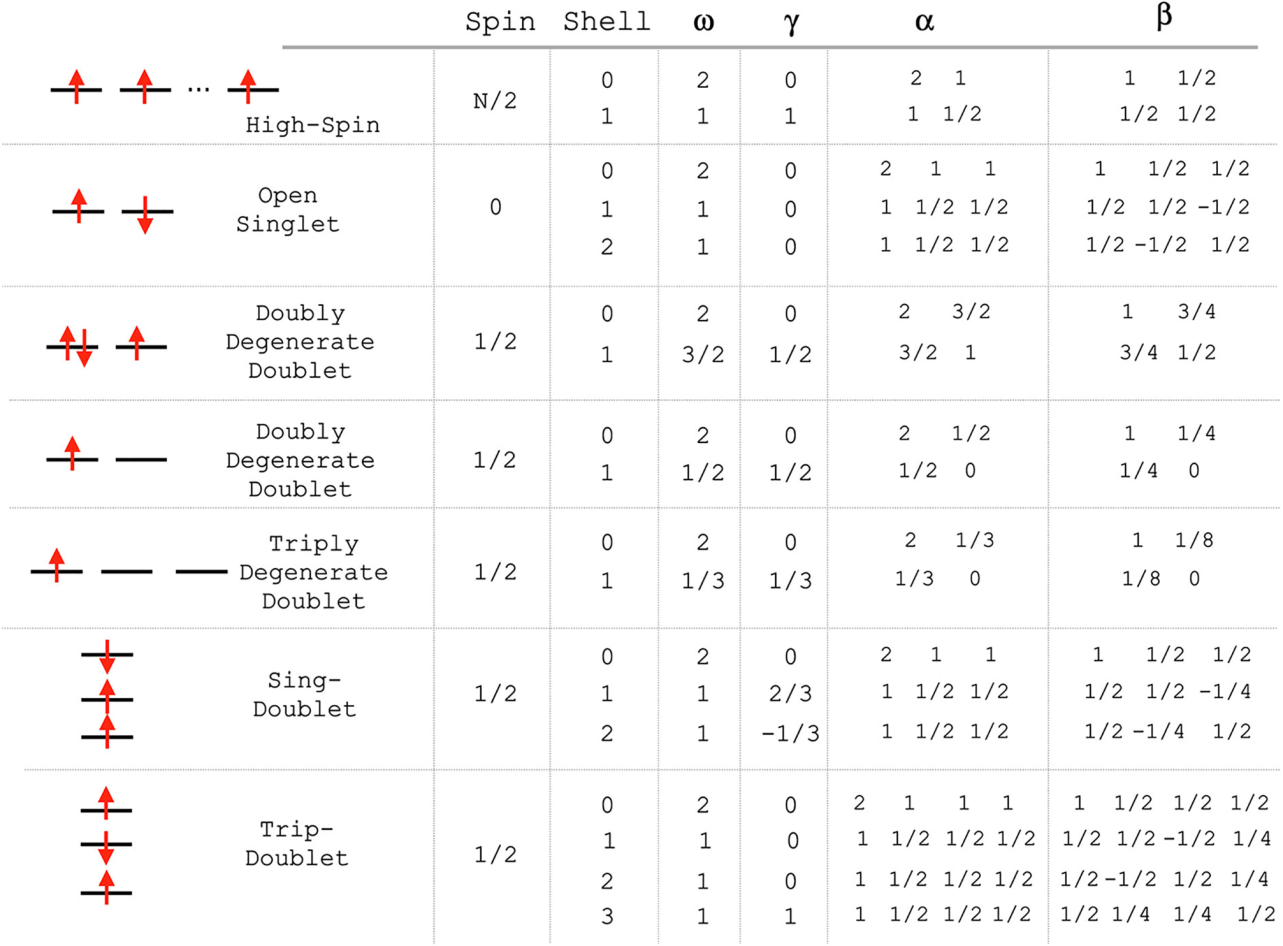
Vector-coupling coefficients for a number of frequently encountered open-shell cases.

For completeness, we should also give the vector-coupling coefficients of Zerner and Edwards for the case of a CAHF wave function for *N* open-shell electrons in *M* open-shell orbitals:^40^

5 $$\begin{array}{l} n_{0}=\omega_{0}=2n_{1}=\omega_{1}=\frac{N}{M}\gamma_{1}=\frac{2S}{M}\\ a_{11}=\frac{2M\left(N-1\right)}{N\left(2M-1\right)}\to \alpha_{11}=\frac{N}{M}\frac{\left(N-1\right)}{\left(2M-1\right)}\\ b_{11}=a_{11}\to \beta_{11}=\frac{1}{2}\frac{N}{M}\frac{\left(N-1\right)}{\left(2M-1\right)} \end{array}$$

All other vector-coupling coefficients in that two-shell system are 1 in the Edwards–Zerner convention.

And the vector-coupling coefficients for a SAHF wave function with *N* open-shell electrons in *M* open-shell orbitals and total spin of *S*:^41^

6 $$\begin{array}{l} n_{0}=\omega_{0}=2n_{1}=\omega_{1}=\frac{N}{M}\gamma_{1}=\frac{2S}{M}\\ a_{11}=\frac{M}{N^{2}\left(M^{2}-1\right)}\left[\mathrm{NM}\left(N-1\right)+\frac{1}{2}N\left(N-4\right)+2S\left(S+1\right)\right]\to \alpha_{11}=\frac{1}{2}{\left(\frac{N}{M}\right)}^{2}a_{11}\\ b_{11}=\frac{M^{2}}{N^{2}\left(M^{2}-1\right)}\left[\frac{2N}{M}\left(N-1\right)+N\left(N-4\right)+4S\left(S+1\right)\right]\to \beta_{11}=\frac{1}{4}{\left(\frac{N}{M}\right)}^{2}b_{11} \end{array}$$

Another highly interesting case is met if one wants to compute the energy of an arbitrary configuration state function given in its branching diagram representation as a list of “+” and “–” couplings. This case was worked out in a previous paper, and the resulting method was called CSF-ROHF.^54^ In this case, the vector-coupling coefficients are given by

7 $$a^{\mathrm{IJ}}=1$$

For any combination of shells. Furthermore

8 $$\begin{array}{l} b^{\mathrm{II}}={2a}^{\mathrm{II}}\\ b^{\mathrm{IJ}}=1-\langle \Psi^{S;N}|E_{u_{J}}^{t_{I}}E_{t_{I}}^{u_{J}}\left|\Psi^{S;N}\right\rangle \end{array}$$

And *n*_0_ = 2, *n*_*I*>0_ = 1. The vector-coupling coefficient γ_*I*_ is discussed below. The number of open-shell operators is given by the number of “kinks” in the branching diagram. Whenever the coupling branch changes direction, a new open shell and corresponding operator are created.

Clearly, the list of wave functions and coupling coefficients given here does not nearly exhaust the list of possibilities of the g-ROHF treatment which underlines the extremely high potential that the method has for the treatment of open-shell molecules.

### 2.2 The Lagrangian and the Self-Consistent Field Solution

In order to start the variational process, we now define the Lagrangian:

9 $$L=V_{\mathrm{NN}}+\sum_{I}\sum_{i_{I}}\omega_{I}h_{i_{I}i_{I}}+\sum_{\mathrm{IJ}}\sum_{i_{I}\;,j_{J}\;}\left\{\alpha_{\mathrm{IJ}}\left(i_{I}i_{I}|j_{J}j_{J}\right)-\beta_{\mathrm{IJ}}\left(i_{I}j_{J}|i_{I}j_{J}\right)\right\}-\sum_{I,J}\sum_{p_{I}\;{,q}_{J}\;}\Lambda_{p_{I}q_{J}}\left(S_{p_{I}q_{J}}-\delta_{p_{I}q_{J}}\right)$$

Where the Lagrange multipliers Λ_*p*_*I*_ *q*_*J*__ ensure orthonormality. Next, the antisymmetric orbital rotation matrix exp­(***κ***) is introduced with *κ*_*p*_*I*_ *q*_*J*__ representing a rotation between two orbitals in shells *I* and *J*, respectively (*I* ≠ *J*).

10 $$S_{p_{I}q_{J}}={\left(c^{T}\mathrm{Sc}\right)}_{p_{I}q_{J}}$$

The overlap matrix in the MO basis, and we have to demand it to be the unit matrix in order to guarantee orthonormality. Since the energy is invariant with respect to intrashell rotations, the number of independent rotation parameters is *N*_*ROT*_ = ∑_*I>J*_ *M*_*I*_ *M*_*J*_, where the virtual shell also must be included.

The orbital rotations are parametrized as

11 C→CU=Cexp⁡(−κ)

For the case of just one open shell, the ***κ***- matrix is of the form:

12 $$\kappa =\left(\begin{array}{lll} 0 & x^{\mathrm{CO}} & x^{\mathrm{CV}}\\ -x^{\mathrm{CO}} & 0 & x^{\mathrm{OV}}\\ -x^{\mathrm{CV}} & -x^{\mathrm{OV}} & 0 \end{array}\right)$$

Where the actual rotation angles *x*_*p*_*I*_ *q*_*J*__^*IJ*^ serve as variational parameters. The general case of an arbitrary number of open shells follows analogously.

It is readily shown that the orbital gradient can be written as

13 $$\frac{\partial E^{\left(K;\mathrm{ROHF}\right)}}{\partial x_{p_{I}q_{J}}}=g_{p_{I}q_{J}}=4\left(F_{p_{I}q_{J}}^{\left(I\right)}-F_{p_{I}q_{J}}^{\left(J\right)}\right)$$

This is consistent with the discussion by Edwards and Zerner.^19^ The shell-specific Fock operators are

14 $$F_{p_{I}q_{J}}^{\left(I\right)}=\frac{1}{2}{\omega_{I}h}_{p_{I}q_{J}}+\sum_{r_{K}}\left\{\alpha_{\mathrm{IK}}\left(p_{I}q_{J}|r_{K}r_{K}\right)-\beta_{\mathrm{IK}}\left(p_{I}r_{K}|q_{J}r_{K}\right)\right\}$$

Which can be re-expressed in the AO basis {μ} as

15 $$F_{\mu \nu }^{\left(I\right)}=\frac{1}{2}{\omega_{I}h}_{\mu \nu }+\sum_{\kappa \tau }\left\{P_{\kappa \tau }^{I;\left(A\right)}\left(\mu \nu |\kappa \tau \right)-P_{\kappa \tau }^{I;\left(B\right)}\left(\mu \kappa |\nu \tau \right)\right\}$$

where we have used the special ‘Coulomb’ and ‘exchange’ densities

16 $$\begin{array}{l} P_{\mu \nu }^{I;\left(A\right)}=\sum_{J}\alpha_{\mathrm{IJ}}P_{\mu \nu }^{\left(J\right)}\\ P_{\mu \nu }^{I;\left(B\right)}=\sum_{J}\beta_{\mathrm{IJ}}P_{\mu \nu }^{\left(J\right)} \end{array}$$

And the shell-projectors

17 $$P_{\mu \nu }^{\left(I\right)}=\sum_{i_{I}}c_{\mu i_{I}}c_{\nu i_{I}}$$

For the virtual shell, we used ***F*** ^(*V*)^ = **0**. The total energy can then be expressed in a compact notation as

18 $$E^{\left(\mathrm{ROHF}\right)}=V_{\mathrm{NN}}+\sum_{I}\left\{\frac{1}{2}\omega_{I}\mathrm{Tr}\left(h,P^{\left(I\right)}\right)+\mathrm{Tr}\left(F^{\left(I\right)},P^{\left(I\right)}\right)\right\}$$

In order to guarantee orthonormal orbitals, it is customary to define a global Fock operator. There is a great deal of flexibility in the definition of this operator. In principle, this combined operator will have the orbital gradient (or something proportional to it) in its off-diagonal, while the intrashell blocks can be defined in any way that benefits the convergence of the self-consistent field procedure. This operator will not play any role for the remainder of the development, but for completeness, one of the possible forms is given as

19 $$F=\sum_{I}P^{\left(I\right)}F^{\left(I\right)}P^{\left(I\right)}+\sum_{I\neq J}P^{\left(I\right)}\left\{F^{\left(I\right)}-F^{\left(J\right)}\right\}P^{\left(J\right)}+\sum_{I}P^{\left(I\right)}F^{\left(I\right)}Q+QF^{\left(I\right)}P^{\left(I\right)}+QF^{\left(0\right)}Q$$

The first term is the intrashell block, the second term defines the off-diagonal elements between two different shells, which is essentially the ROHF orbital gradient, the third off-diagonal between an occupied and a virtual orbital, and the last term defines the virtual space (***Q*** = **1** – ∑_*I*_ ***P*** ^(*I*)^ is the projector onto the virtual space). A stationary point of the ROHF solution is reached when the off-diagonal blocks of the global Fock matrix are all zero, which is the case when the orbital gradient vanishes.

The Lagrange multiplier matrix is

20 $$\Lambda =2\sum_{I}P^{\left(I\right)}F^{\left(I\right)}P^{\left(I\right)}$$

Note that the virtual block of this matrix is zero.

The total density is

21 $$P=\sum_{I}\omega_{I}P^{\left(I\right)}$$

The spin density is interesting because, as discussed above, each shell will contribute in a way that depends on the structure of the wave function. We may write

22 $$R=\sum_{I}\gamma_{I}P^{\left(I\right)}$$

If *n*_σ_^*I*^ is the occupation number of shell *I* with spin σ = α, β, then γ_*I*_ = *n*_σ_^*I*^ – *n*_β_^*I*^ is the spin density coupling coefficient (obviously ω_*I*_ = *n*_*I*_ = *n*_α_^*I*^ + *n*_β_^*I*^). Clearly, the closed shell does not contribute to the spin density, and hence γ_0_ = 0.

23 $$n_{\sigma }^{I}=\frac{1}{M_{I}}\sum_{p_{I}}\left\langle \Psi \left|a_{p\sigma }^{+}a_{p\sigma }\right|\Psi \right\rangle$$

### 2.3 Analytic Nuclear Gradients

Given the SCF solution, the calculation of the nuclear gradients is straightforward. The derivative with respect to a nuclear coordinate *X*_*M*_ *is:*

24 $$\frac{\partial L}{{\partial X}_{M}}=\frac{\partial V_{\mathrm{NN}}}{{\partial X}_{M}}+\sum_{\mu \nu }P_{\mu \nu }\frac{\partial h_{\mu \nu }}{\partial X_{M}}+\frac{1}{2}\sum_{\mu \nu \kappa \tau }\left\{P_{\mu \nu }P_{\kappa \tau }^{\left(A\right)}-P_{\mu \kappa }^{\left(B\right)}P_{\nu \tau }^{\left(B\right)}\right\}\frac{\partial \left(\mu \nu |\kappa \tau \right)}{\partial X_{M}}+\sum_{\mu \nu }W_{\mu \nu }\frac{{\partial S}_{\mu \nu }}{{\partial X}_{M}}$$

With the energy weighted density simply given as

$$W=c\Lambda c^{T}$$ 25

This equation is implemented in the ORCA program package but will not be further discussed in the remainder of the paper.

### 2.4 The Orbital Hessian

It is a somewhat tedious yet elementary exercise to derive the second derivatives of the energy. They read:

26 ∂2LxpIqJxrKsL≡(A+B)pIqJ,rKsL=4{δIKδrKpI(FsLqJ(I)−FsLqJ(J))−δILδsLpI(FrKqJ(I)−FrKqJ(J))+δJKδrKqJ(FpIsL(I)−FpIsL(J))−δJLδsLqJ(FpIrK(I)−FpIrK(J))}+8(αIK+αJL−αIL−αJK)(pIqJ|rKsL)−4(βIK+βJL−βIL−βJK){(pIrK|qJsL)+(pIsL|qJrK)}

This equation was carefully verified by direct numerical differentiation of the ROHF energy. A closely related, but not identical, equation was given by Fernandez–Rico et al. in refs 34,35 .

In order to demonstrate that this equation correctly reduces to the familiar closed-shell orbital Hessian. The relevant data can be inserted, noting that in this case, there is only a closed (“*O*”) and a virtual (“*V*”) shell. Assuming a diagonal Fock operator, one obtains:

$${\left(A+B\right)}_{p_{I}q_{J},r_{K}s_{L}}=4\delta_{\mathrm{ia},\mathrm{jb}}\left(\varepsilon_{a}-\varepsilon_{i}\right)+16\left(\mathrm{ia}|\mathrm{jb}\right)-4\left\{\left(\mathrm{ij}|\mathrm{ab}\right)+\left(\mathrm{ib}|\mathrm{aj}\right)\right\}$$

$$\alpha_{\mathrm{IJKL}}=\alpha_{\mathrm{OVOV}}=8\left(\alpha_{\mathrm{OO}}+\alpha_{\mathrm{VV}}-\alpha_{\mathrm{VO}}-\alpha_{\mathrm{OV}}\right)=8\alpha_{\mathrm{OO}}=16$$

$$\beta_{\mathrm{IJKL}}=4\left(\beta_{\mathrm{OO}}+\beta_{\mathrm{VV}}-\beta_{\mathrm{VO}}-\beta_{\mathrm{OV}}\right)=4$$

27 $$4\left\{\delta_{\mathrm{ij}}\left(F_{\mathrm{ab}}^{\left(I\right)}\right)-\delta_{\mathrm{JL}}\delta_{\mathrm{ab}}\left(F_{\mathrm{ij}}^{\left(I\right)}\right)\right\}=4\left(\varepsilon_{a}-\varepsilon_{i}\right)$$

This is obviously just four times the result that is usually quoted for the response matrix in RHF theory (α_*OO*_ = 2, α_*VV*_ = 0, β_*OO*_ = 1, β_*VV*_ = 0, and *F*_*pq*_^(*V*)^ = 0). However, the factor of 4 appears in identical form on both sides of the coupled-perturbed SCF (CP-SCF) equations and is usually eliminated in actual applications.

### 2.5 The Magnetic Hessian

For magnetic perturbations, we need to derive the magnetic Hessian. For such a perturbation, the Coulomb term vanishes and we get

28 (A−B)pIqJ,rKsL=4{δIKδrKpI(FsLqJ(I)−FsLqJ(J))−δILδsLpI(FrKqJ(I)−FrKqJ(J))+δJKδrKqJ(FpIsL(I)−FpIsL(J))−δJLδsLqJ(FpIrK(I)−FpIrK(J))}−4(βIK+βJL−βIL−βJK){(pIrK|qJsL)−(pIsL|qJrK)}

### 2.6 The Coupled-Perturbed ROHF Equations for the Real Perturbations

We are now equipped to write down the coupled-perturbed g-ROHF equations. To this end, we add the term λ*V* ^(λ)^ to the Lagrangian.

29 L(λ)=VNN+∑I∑iIωI∑μν⟨μ|h+λV(λ)|ν⟩Pμν(I)+∑μν∑IPμν(I)∑κτ{Pμν(I;A)(μν|κτ)−Pμν(I;B)(μτ|κν)}−∑IJ∑pIqJΛpIqJ(∑μνSμνcμpIcνqI−δpIqJ)

And we need to expand everything in terms of λ including the rotation angles *x*_*p*_*I*_ *q*_*J*__ (λ). The perturbing operator *V* ^(λ)^ might as well be absorbed into the one-electron operator *h*, recognizing that the derivative of $h^{\left(\lambda \right)}=\frac{\partial h}{\partial \lambda }$ then contains it. Intrashell rotations are determined from the condition:

30 $$\sum_{\mu \nu }S_{\mu \nu }c_{\mu p_{I}}c_{\nu q_{J}}=\delta_{p_{I}q_{J}}$$

yielding:

31 $$S_{p_{I}q_{J}}^{\left(\lambda \right)}=-x_{q_{J}p_{I}}^{\left(\lambda \right)}-x_{p_{I}q_{J}}^{\left(\lambda \right)}$$

Hence, for real perturbations, we can choose the rotation angles for the intrashell rotations to be

32 $$x_{q_{I}p_{I}}^{\left(\lambda \right)}=x_{p_{I}q_{I}}^{\left(\lambda \right)}=-\frac{1}{2}S_{p_{I}q_{I}}^{\left(\lambda \right)}$$

Following some algebra, one arrives at the coupled-perturbed g-ROHF equations in the familiar form:

33 $$\left(A+B\right)U^{\left(\lambda \right)}=-b^{\left(\lambda \right)}$$

where the factor of 4 in eq 26 has been dropped. This allows one to express the right-hand-side as

34 bpIqJ(λ)=12(ωI−ωJ)VpIqJ(λ)−(ΛS(λ)+S(λ)Λ)pIqJ+(GpIqJ(I)(−12S(I;λ))−GpIqJ(J)(−12S(J;λ)))+(FpIqJ(I;AOD)−FpIqJ(J;AOD))

where ***G*** ^(*I*)^ is the two-electron part of ***F*** ^(*I*)^ and ***F*** ^(*I,AOD*)^ is the Fock operator formed with derivative integrals. ***S*** ^(λ)^ are the derivative overlap integrals transformed into the MO basis, and ***S*** ^(*I*;λ)^ represent the derivative of the overlap integrals projected into the MO’s of only shell *I*.

Clearly, an analogous equation holds for purely imaginary Hermitian perturbation by substituting the **(** ***A*** **–** ***B*** **)** matrix for **(** ***A*** **+** ***B*** **)** (and also dropping the factor of 4 from both sides of the equation).

### 2.7 Instabilities in the Response Equations

It turns out that the response matrices can have small negative eigenvalues even for perfectly valid g-ROHF solutions with zero orbital gradients. A detailed analysis of the corresponding eigenvectors reveals that they often correspond to symmetry-breaking rotations. As discussed in more detail by Crawford and coauthors,^57,58^ this symmetry breaking can frequently be considered to be unphysical. Clearly, the negative eigenvalues can have adverse effects on the calculation of the response properties. This is most readily seen by rewriting the equations in terms of the spectral resolution of the response matrix. One can write ***R*** = (***A*** **+** ***B***) as an operator in terms of its eigenvalues *r*_*x*_ and eigenvectors **|** ***X*** ⟩ as

35 $$\mathrm{R̂}=\sum_{X}|X\rangle r_{x}\langle X|$$

And the inverse operator

36 $${\mathrm{R̂}}^{-1}=\sum_{X}|X\rangle \frac{1}{r_{x}}\langle X|$$

Which means that the solution to the CP-SCF equations can be written as

37 $$U^{\left(\lambda \right)}=-\sum_{X}\frac{|X\rangle \langle X|}{r_{x}}b^{\left(\lambda \right)}$$

Thus, a real-valued second-order property can be expressed as

38 $$\alpha_{\kappa \lambda }=2U^{\left(\kappa \right)}b^{\left(\lambda \right)}=-2\sum_{X}\frac{\langle b^{\left(\kappa \right)}|X\rangle \left\langle X|b^{\left(\lambda \right)}\right\rangle }{r_{x}}$$

Which is a sum-over-states (SOS) like representation of the second-order property that is mathematically equivalent to the solution of the response equations. In this formulation, the eigenvectors of the response matrix serve as the excited states of the system, and *r*_*x*_ serves as the excitation energies. In fact, in the Tamm–Dancoff approximation, this is exactly how the excited states of the system are calculated, and consequently, the solution of the response equations is equivalent to an untruncated SOS.

Given these relationships, it becomes clear why small and negative eigenvalues of the response matrix are potentially harmful for the calculation of the response properties. They will lead to divergence of the response equation or ill-conditioned linear equations. As long as one can argue that the corresponding offending eigenvectors are unphysical and represent undesired symmetry-breaking modes, one can eliminate them by solving the modified response problem:

39 $$\mathrm{Q̃R}\mathrm{Q̃}U^{\left(\lambda \right)}=-b^{\left(\lambda \right)}$$

where in this context ***Q̃*** **= 1 –** ***P̃*** is a projector onto the orthogonal complement of the undesired “outer” space spanned by the corresponding eigenvectors ***P̃*** = ∑_*X,r*_*x*_ ≤ 0_ **|** ***X*** **⟩⟨** ***X*** **|**. Depending on the solver used for solving the response equations, it may be desirable to also shift the eigenvalues of the offending eigenvectors by a large number ζ (e.g., 10^6^ Eh) by replacing ***Q̃RQ̃*** by ***Q̃RQ̃*** + ζ ***P̃***. An example will be studied in the numerical section below.

## 3 Implementation

In order to implement the response equations derived above, an atomic-orbital (AO) driven procedure was developed. Since the equations are high-dimensional for larger molecules, an iterative approach is usually followed in which the key step is the formation of the σ-vector:

σ=Rt 40

Where ***R*** (e.g., ***A*** **±** ***B***) is the response matrix and ***t*** a trial vector. The most straightforward way of implementation proceeds by transforming the trial vector block by block into the AO basis

41 $$t_{\mu \nu }^{\mathrm{IJ}}=\sum_{p_{I}\;q_{J}\;}c_{\mu p_{I}}c_{\nu q_{J}}t_{p_{I}q_{J}}$$

One can then form the Coulomb and exchange operators in the AO basis:

42 $$J_{\mu \nu }^{\mathrm{IJ}}=\sum_{\kappa \tau }t_{\kappa \tau }^{\mathrm{IJ}}\left(\mu \nu |\kappa \tau \right)$$

43 $$K_{\mu \nu }^{\mathrm{IJ}}=\sum_{\kappa \tau }t_{\kappa \tau }^{\mathrm{IJ}}\left(\mu \kappa |\nu \tau \right)$$

That are then transformed back into the MO basis, and added to the σ-vector with their appropriate prefactors (note that eq 26 has been divided by a factor of 4 in line with the discussion above):

44 $$\sigma_{p_{I}q_{J}}=\sigma_{p_{I}q_{J}}^{\left(\mathrm{Coulomb}\right)}+\sigma_{p_{I}q_{J}}^{\left(\mathrm{exchange}\right)}+\sigma_{p_{I}q_{J}}^{\left(\mathrm{Fock}\right)}$$

45 $$\sigma_{p_{I}q_{J}}^{\left(\mathrm{Coulomb}\right)}=\sum_{K>L}\alpha_{\mathrm{IJKL}}J_{p_{I}q_{J}}^{\mathrm{KL}}$$

46 $$\sigma_{p_{I}q_{J}}^{\left(\mathrm{exchange}\right)}=-\sum_{K>L}\beta_{\mathrm{IJKL}}\left(K_{p_{I}q_{J}}^{\mathrm{KL}}+K_{{q_{J}p}_{I}}^{\mathrm{KL}}\right)$$

47 $$\sigma_{p_{I}q_{J}}^{\left(\mathrm{Fock}\right)}=\delta_{\mathrm{IK}}\delta_{r_{K}p_{I}}\left(F_{s_{L}q_{J}}^{\left(I\right)}-F_{s_{L}q_{J}}^{\left(J\right)}\right)-\delta_{\mathrm{IL}}\delta_{s_{L}p_{I}}\left(F_{r_{K}q_{J}}^{\left(I\right)}-F_{r_{K}q_{J}}^{\left(J\right)}\right)+\delta_{\mathrm{JK}}\delta_{r_{K}q_{J}}\left(F_{{p_{I}s}_{L}}^{\left(I\right)}-F_{{p_{I}s}_{L}}^{\left(J\right)}\right)-\delta_{\mathrm{JL}}\delta_{s_{L}q_{J}}\left(F_{p_{I}r_{K}}^{\left(I\right)}-F_{p_{I}r_{K}}^{\left(J\right)}\right)$$

48 $$\alpha_{\mathrm{IJKL}}=2\left(\alpha_{\mathrm{IK}}+\alpha_{\mathrm{JL}}-\alpha_{\mathrm{IL}}-\alpha_{\mathrm{JK}}\right)$$

49 $$\beta_{\mathrm{IJKL}}=\left(\beta_{\mathrm{IK}}+\beta_{\mathrm{JL}}-\beta_{\mathrm{IL}}-\beta_{\mathrm{JK}}\right)$$

Note that the magnetic Hessian differs from the electric one only by changing the sign of *K*_*p*_*I*_ *q*_*J*__^*KL*^ + *K*_*q*_*J*_ *p*_*I*__^*KL*^ in the exchange term to *K*_*p*_*I*_ *q*_*J*__^*KL*^ + *K*_*q*_*J*_ *p*_*I*__^*KL*^ and the omission of the Coulomb term. Likewise, the formation of sigma vectors for excitation energy calculations within the random-phase approximation (RPA) or Tamm–Dancoff (TDA) formalisms is trivial, as will be explored in a forthcoming paper.

Given a solution vector ***t*** ^(λ)^, we can form the AO basis response electron (***P*** ^(λ)^) and spin (***R*** ^(λ)^) densities:

50 $$\begin{array}{l} P_{\mu \nu }^{\left(\lambda \right)}=\sum_{I>J}\left(\omega_{I}-\omega_{J}\right)\sum_{p_{I}\;q_{J}\;}c_{\mu p_{I}}c_{\nu q_{J}}t_{p_{I}q_{J}}\\ R_{\mu \nu }^{\left(\lambda \right)}=\sum_{I>J}\left(\gamma_{I}-\gamma_{J}\right)\sum_{p_{I}\;q_{J}\;}c_{\mu p_{I}}c_{\nu q_{J}}t_{p_{I}q_{J}} \end{array}$$

As explained elsewhere,^59^ these response densities are stored in a central density container to be used by the property program of the ORCA package for the calculation of response properties. Thus, all properties that ORCA can calculate are automatically available for g-ROHF following the implementation of the response equations and the formation of the response densities.

We note that in our implementation, the AO basis Coulomb operators can either be formed using exact four-index repulsion integrals or within the Split-RI-J approximation^60,61^ as well as the recently proposed linear-scaling RI-BUPO/J formalism.^62^ Likewise, exchange matrices can be formed either with exact four-index integrals or using the very efficient chain-of-spheres (COSX) algorithm.^63–65^ Solvation terms from the CPCM model can also be considered.

As discussed above, small negative eigenvalues in the response treatment can adversely affect the calculation of the response properties in the general ROHF formalism. The program has therefore been set up in a way that, prior to the solution of the response equations, the lowest eigenvalues and vectors of the corresponding response matrix can be determined using a Davidson algorithm. If negative eigenvalues are found, then the corresponding vectors will be used to project the response equations. This can be accomplished by projecting any component of the trial and sigma vectors along the direction of the offending eigenvectors.

## 4 Numerical Results

In this section, a few numerical results are assembled. We start with a selection of 15 small open-shell molecules, some of which feature an orbitally degenerate ground state. We then move on to discuss the orbital instabilities in O_2_ and the peculiarities of the g-tensors of orbitally degenerate molecules. The calculation of hyperfine couplings, in particular the spin–orbit coupling contribution to the metal hyperfine, is demonstrated with the well-studied case of [Cu­(NH_3_)_4_]^2+^. Finally, in order to demonstrate the generality of the proposed methodology, a few selected cases with more complicated spin–coupling patterns are investigated.

### 4.1 Small Molecule g-Tensors and Polarizabilities

As a first test of the method, a set of 15 small open-shell species was investigated, and their isotropic polarizabilities and g-values were calculated as representative properties in the class of real and imaginary perturbations. The geometries of all species were optimized at the CCSD­(T)/cc-pVTZ level. All property calculations employed the def2-QZVPP basis set. It is noteworthy that all species with an orbitally nondegenerate ground state (^2^Σ, ^3^Σ, ^2^A_1_, ^2^A′, ^2^B_2_) are standard ROHF cases. However, the ^2^Π species in the test set represent nonstandard cases that require the g-ROHF treatment. These species will break symmetry in UHF-based calculations with disastrous consequences for their magnetic response properties, as will be discussed below.

The polarizabilities were referenced against spin-unrestricted CCSD calculations. The results collected in Table 2 show that the ROHF and UHF polarizabilities are nicely consistent. Overall, the ROHF calculations agree slightly better with the CCSD reference data as evidenced by the smaller mean deviation, mean absolute deviation, and also maximum deviation. The cases with the largest deviations in the UHF calculations are also the most strongly spin-contaminated.

**Table 2:** Geometries and Isotropic Polarizabilities (in Atomic Units) for a Set of 15 Small Open-Shell Species^a^

molecule	state	geometry	<S^2^>_UHF_	UHF	ROHF	UCCSD
CN	^2^∑^–^	*R* = 1.745	1.171	14.102	17.524	17.683
CO^+^	^2^∑^–^	*R* = 1.1192	0.983	8.263	8.278	8.472
BO	^2^∑^–^	*R* = 1.2134	0.802	14.968	14.858	15.859
BeH	^2^∑^+^	*R* = 1.3385	0.752	31.857	31.690	31.945
BH	^2^∑^+^	*R* = 1.2065	0.755	9.982	9.849	10.011
CH	^2^Π	*R* = 1.1224	0.760	12.542	12.426	12.571
NH	^3^∑^–^	*R* = 1.0392	2.017	8.232	8.326	8.392
OH	^2^Π	*R* = 0.9711	0.757	5.589	5.551	5.887
FH^+^	^2^Π	*R* = 1.0017	0.755	2.600	2.587	2.732
NO	^2^Π	*R* = 1.1530	0.804	10.026	9.860	9.946
O_2_	^3^∑_g_^–^	*R* = 1.2122	2.048	10.012	10.887	9.060
H_2_O^+^	^2^A_1_	*R*_OH_ = 1.0012 *A*_HOH_ = 108.861	0.758	4.887	4.894	5.059
HCO	^2^A′	*R*_CH_ = 1.209 R_CO_ = 1.1824 *A*_HCO_ = 124.363	0.766	15.256	14.963	15.683
NO2	^2^B_2_	*R*_NO_ = 1.993 *A*_ONO_ = 134.200	0.814	16.076	16.667	14.886
H_2_CO^+^	^2^B_2_	*R*_CO_ = 1.200 R_CH_ = 1.1135 *A*_HOC_ = 119.399	0.786	11.622	11.398	12.204
*MD*			*–0.292*	*–0.042*		
*MAD*			*0.588*	*0.523*		
*MAX*			*3.581*	*1.828*		

a The electronic ground state and UHF spin-expectation values are provided. All calculations were done with the def2-QZVPP basis set. Statistics (MD = mean deviation, MAD, mean absolute deviation, MAX = maximum deviation) are references against unrestricted CCSD calculations, including orbital relaxation.

For magnetic response properties, the situation is quite different. Here, one has to exclude the ^2^Π species from the statistics since the unrestricted calculations, no matter whether they are done at the UHF or CCSD level, provide unphysical results because they cannot handle the orbital degeneracy properly. Since this subject is interesting, it is further discussed in the next section. The remaining orbitally nondegenerate species are referenced against large-scale MRCI+Q calculations that were done according to the method described in refs 66,67 employing an uncontracted and individually selecting MRCI scheme. The orbitals were optimized with a full-valence SAHF method, and MRCI calculations used a full-valence CAS space in all cases. The prediagonalization (T_pre_) and selection (T_sel_) thresholds were set to tight values of 1e-4 and 1e-10 Eh, respectively, except for NO_2_ and H_2_CO^+^, where the selection threshold was set to 1e-3. The number of roots was adjusted such that all states up to about 1,00,000 cm^–1^ above the ground state are covered in the MRCI calculation, which required <20 roots to be calculated in all cases. In all calculations, the full SOMF operator^68,69^ was used to represent the SOC.

The data collected in Table 3 do not show a particularly clear trend. For the cases where CCSD is applicable, the MRCI and CCSD data are in reasonable agreement. Unfortunately, it is not really possible to decide which data set is more accurate because the sparse experimental data that exist for such species are inconclusive and are often also influenced by the fact that data are taken in inert gas matrices rather than the gas phase. For H_2_O^+^, one of the few species for which gas-phase EPR has been measured, the CCSD value is a little closer to experiment than MRCI+Q but both values differ significantly from the experimentally reported value. The MRCI+Q g-shifts are uniformly smaller than the CCSD ones. Whether this is due to missing orbital relaxation, the truncation of the sum-overstates expansion in the MRCI+Q calculation, or perhaps even represents a superior result is uncertain. The statistics for MRCI+Q are compromised by the value for H_2_CO^+^ that is far off from the other methods. It is, however, much closer to the reported experimental value of 1333 ppm (quoted from ref 12) than any of the other methods. Without H_2_CO^+^ MRCI+Q and CCSD are much closer, with a mean absolute deviation of about 300 ppm, from each other.

**Table 3:** Geometries and Isotropic g-Tensors (Ppm) for a Set of 15 Small Open-Shell Species^a^

molecule	state	<S^2^>_UHF_	UHF	ROHF	*MRCI+Q*	UCCSD
CN	^2^∑^–^	1.171	*-1449.6*	*-797.3*	*-1300.9*	*-1883.4*
CO^+^	^2^∑^–^	0.983	*-2138.7*	*-946.0*	*-1574.5*	*-2339.2*
BO	^2^∑^–^	0.802	*-1372.9*	*-542.5*	*-1079.5*	*-1478.0*
BeH	^2^∑^+^	0.752	*-101.4*	*-95.5*	*-104.6*	*-116.2*
BH	^2^∑^+^	0.755	*-475.7*	*-469.1*	*-484.4*	*-557.7*
CH	^2^Π	0.760	*-7874.0*	152.6	*-2000777.3*	*-90745.0*
NH	^3^∑^–^	2.017	645.4	855.3	702.4	918.3
OH	^2^Π	0.757	28427.4	2686.8	*-668105.8*	436465.0
FH^+^	^2^Π	0.755	58097.5	10704.5	*-667762.9*	1186244.2
NO	^2^Π	0.804	*-42683.4*	980.2	*-1999318.4*	*-267177.4*
O_2_	^3^∑_g_^–^	2.048	2180.9	2062.9	1635.6	1976.8
H_2_O^+^	^2^A_1_	0.758	5095.1	6180.6	6046.9	6811.5
HCO	^2^A’	0.766	*-1833.2*	*-1162.1*	*-1792.1*	*-1833.2*
NO2	^2^B_2_	0.814	*-3072.4*	*-2223.7*	*-2260.7*	*-2401.1*
H_2_CO^+^	^2^B_2_	0.786	2911.1	3440.5	1858.0	3751.6
*MD*			*-253.7*	*-227.4*	*-475.2*	
*MAD*			412.9	559.1	475.2	
*MAX*			1716.4	1192.7	1893.6	

a The electronic ground state and UHF spin-expectation values are provided. All calculations were done with the def2-QZVPP basis set. The statistical evaluation excludes the ^2^Π systems and uses the CCSD reference data.

Relative to the CCSD data, both the UHF and the ROHF response methods have a tendency to underestimate the g-shifts. That trend is, however, not uniform, as ROHF overestimates the g-shifts of NH, O_2_, and H_2_O^+^. Overall, the ROHF and UHF responses are of comparable quality relative to the reference data. The UHF method has a slightly lower mean absolute deviation, whereas the ROHF method has a lower maximum deviation. Clearly, both methods are of moderate accuracy for this particular property.

### 4.2 Instability Issues for O_2_

The case of the O_2_ (^3^∑^–^) molecule is interesting and deserves a more detailed investigation. In this case, the ROHF equations smoothly converge to the desired ^3^∑^–^ ground state in which the two π* orbitals are each singly occupied with parallel spin electrons. Despite the fact that the O_2_ molecule has no low-lying electronic excited states, the A-matrix shows a pair of degenerate negative eigenvalues at about −0.003 Eh, indicating that the solution is, in fact, a saddle point. In order to investigate this further, some code was written that allows for making finite displacements along the offending eigenvectors followed by re-evaluation of the energy. This is accomplished by a Cayley transformation. Let ***v*** ^(*I*)^ (*I* = 0, 1) be an eigenvector of ***A*** in rotation angle space. We can then apply a transformation to the MO coefficient matrix ***c*** ^(ref)^ at the reference point to obtain a new coefficient matrix ***c*** (*x*) that depends on a finite displacement *x* along ***v*** ^(*I*)^. To this end, form the matrix of rotation angles

51 $$K_{p_{I}q_{J}}\left(x\right)=\{\begin{array}{l} xv_{p_{I}q_{J}}^{\left(I\right)}p_{I}>q_{J}\\ -xv_{p_{I}q_{J}}^{\left(I\right)}p_{I}<q_{J} \end{array}$$

and solve the linear equation system

52 $$\left(1+\frac{1}{2}K\left(x\right)\right)U\left(x\right)=1-\frac{1}{2}K\left(x\right)$$

To get the new MO coefficients

53 $$c\left(x\right)=c^{\left(\mathrm{ref}\right)}U\left(x\right)$$

These coefficients are then used to calculate the corresponding density matrices (eqs 16 and 17) followed by the evaluation of the Fock matrix (eq 15) energy (eq 18). We note in passing that a similar approach was used to numerically verify the correctness of the response equations.

The results shown in Figure 2 demonstrate clearly that the stationary point found is indeed a saddle point and that there is a continuum of minima at a displacement of roughly 0.07 along both offending modes, forming a Mexican hat kind of potential energy surface with a minimum that is about −400 micro-Eh below the SCF solution.

**Figure 2:**
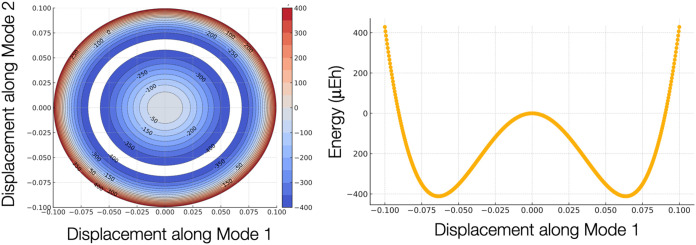
Potential energy surface of the ROHF ^3^∑^−^ ground state of O_2_/def2-SVP as a function of displacement along the two eigenvectors belonging to negative eigenvalues of the response matrix.

Inspection of the eigenvectors shows that they mostly feature rotations between the doubly occupied π–orbitals and the singly occupied π*–orbitals. While this initially may indicate a poorly converged SCF solution, inspection of the orbital gradient shows that this is not the case, and the orbital gradient is zero to machine precision at the SCF reference point. Plotting the orbitals at the SCF solution reference point and at the manually located minimum does, however, not reveal any peculiar symmetry-breaking shape (Figure 3). Thus, these small negative eigenvalues of the response matrix, even in electronic situations where there is no obvious near degeneracy, appear to be a feature one has to be aware of in ROHF calculations because they may severely affect the outcome of response calculations. For O_2_, this is, however, not the case, and the polarizabilities and g-tensors are identical for projected and unprojected response equation solutions. According to eq 38, this will be the case if the projection of the right-hand side vectors onto the offending eigenvector of the A-matrix vanishes.

**Figure 3:**
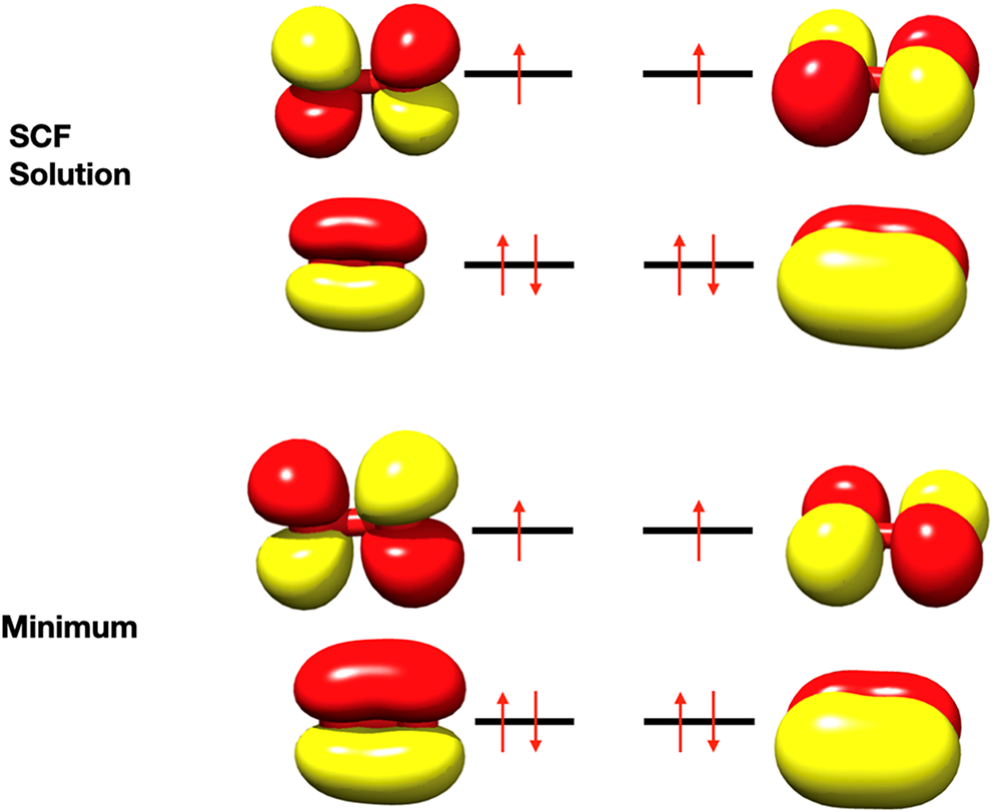
π and π* orbitals of the π- and π*-orbitals of the O_2_/def2-SVP at the SCF solution and at the energy minimum of Figure 2.

### 4.3 The g-Tensors for Molecules with Degenerate Ground States

An interesting case is met in the orbitally degenerate molecules with a ^2^Π ground state that can be thought of as having components ^2^Π_*x*_ and ^2^Π_*y*_. These molecules are subject to first-order SOC, which will consequently dominate their magnetic properties. One can write the matrix of the SOC operator in the basis of the four magnetic sublevels of the ^2^Π state as ( $M=\pm \frac{1}{2}$ )­

54 ⟨Π2IM|ĤBO+ĤSOC|Π2JM′⟩→(00+i2ζ0000−i2ζ−i2ζ00+i2ζ0000)

where ζ is the effective SOC constant (that can be positive or negative depending on the electronic configuration). One readily finds the eigenvalues of this matrix to be $\mp \frac{\zeta }{2}$ with the eigenfunctions:

55 |Π21/2M⟩=12(|Π2xM⟩+|Π2yM⟩)|Π23/2M⟩=i2(|Π2xM⟩−|Π2yM⟩)

From this, one can readily deduce that the Kramers pair |^2^Π_1/2_^*M*^⟩ has *g*-values *g* = 0,0,0 whereas the Kramers pair |^2^Π_3/2_^*M*^⟩ has *g*-values *g* = 0,0,4. Hence, |^2^Π_1/2_^*M*^⟩ will be EPR-silent and |^2^Π_3/2_^*M*^⟩ will have a highly anisotropic EPR spectrum.

UHF-based electronic structure methods cannot describe such a state properly. They will arbitrarily occupy one of the components (or a linear combination thereof) and leave the other unoccupied. This will lead to symmetry breaking and will later show up in the response treatment as very large and erratic responses to external perturbations that are a result of the broken degeneracy. The disastrously wrong results for the *g*-values of the ^2^Π molecules in Table 3 clearly illustrate this situation.

The g-ROHF method, on the other hand, will keep the degeneracy intact. Since the energy is invariant with respect to rotations of orbitals that are in the same shell, the response will contain *no* component in the open active shell. This situation creates a unique opportunity: the response of the g-ROHF method will capture all the interactions of the orbitally degenerate ground state (here ^2^Π) with all other excited states of the system that couple to it via SOC (or any other perturbation). These are second-order effects. At the same time, the first-order effects can be captured with a minimal CI calculation that only includes the degenerate or quasi-degenerate components. The sum of the two contributions will then define the final result for the tensor.

This construction proposes a solution to an old problem of QDPT theory, namely, that it is effectively a truncated SOS expansion where the included states are treated to infinite order while the remaining states, and in particular those outside the active space, are not treated at all. With the combination of QDPT and g-ROHF response theory, one can consider having the best of both worlds: the efficiency and simplicity of the response treatment with the robustness and rigor of the QDPT treatment. In Scheme 1, a simple input file is shown that illustrates how to implement this combination of response and QDPT theory in the ORCA program.

**Scheme 1:**
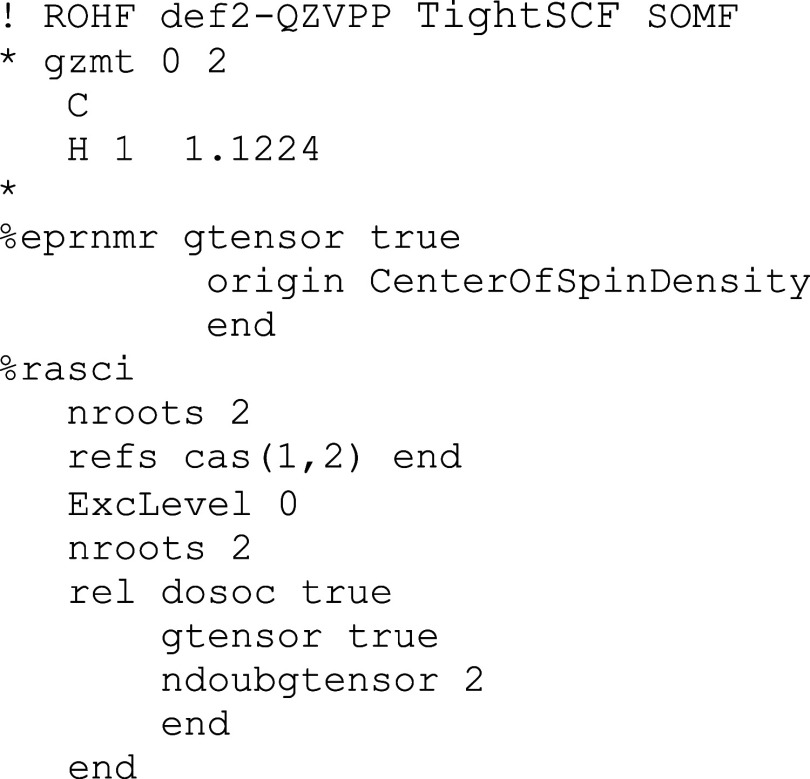
Illustration of How the Combination of Response and QDPT Theory Can Be Achieved in an ORCA Input Fort the CH Radical in a ^2^Π Ground State (ORCA Input Syntax)

It is emphasized that this strategy does not only hold for doubly degenerate states or for exactly degenerate states. It will always be applicable in situations in which the quasi-degenerate space can be captured within one shell of the g-ROHF method. Hence, near degeneracy can also be treated with this approach. For example, one can use CAHF or SAHF to average over the nearly degenerate components and then employ g-ROHF to calculate the second-order terms together with a very small CI expansion followed by QDPT to capture the first-order contributions.

For the case at hand, the first-order contribution to the g-tensor of the ^2^Π molecules is so overwhelmingly large that it is essentially pointless to add a second-order contribution. This, however, is not a universal conclusion and will not hold for systems with weak in-state SOC or comparatively large splittings between the quasi-degenerate components.

### 4.4 g-Tensors and Hyperfine Couplings in Transition-Metal Complexes: [Cu­(NH_3_)_4_]^2+^

In order to demonstrate that the new code is able to calculate hyperfine couplings, including the SOC correction to it, a widely used model system—[Cu­(NH_3_)_4_]^2+^ has been reinvestigated.^70^ This is a *d* ^9^ system with a single unpaired electron in the copper d-shell that occupies the highly antibonding *d*_x2‑y2_ based molecular orbital. The physics of EPR parameters of Cu­(II) has been discussed many times before.^12,13,66,70–72^ It suffices to recall that the quantum chemical calculation of these parameters has been found to be particularly difficult since the delicate balance between predicting the correct spin-distribution (e.g., the metal–ligand covalency) and the ligand-field excitation energies must be met. In addition, the rather intricate core-level spin-polarization is essential for the correct prediction of the isotropic metal hyperfine coupling.^71,73,74^ The so far best results have been obtained with the spectroscopy-oriented configuration interaction (SORCI)^75^ method, an uncontracted and individually selected multireference-CI method.^70^

In this work, the X2C Hamiltonian^76^ was used together with the X2C-SVPall basis set.^77,78^ This is a relatively small basis set that will not yield fully converged results, but it will allow for the application of the calculation of the EPR parameters at the CCSD level.

The results in Table 4 show the expected results. The g-shifts are massively overestimated by both the UHF and ROHF approaches, which is expected because the description of the bonding delivered by Hartree–Fock methods is far too ionic and places more than 90% of the spin population on the copper center, thus leading to a strong exaggeration of the SOC contribution to the g-tensor. The isotropic metal-hyperfine coupling is also very poorly represented by either ROHF (where spin-polarization is absent by construction) or UHF (which massively overestimates spin-polarization). As expected, the nitrogen hyperfine couplings are underestimated by either UHF or ROHF, which is a result of insufficient spin-delocalization onto the ligand nuclei. Here, however, UHF does a little better than ROHF. The SOC contribution to the metal hyperfine coupling is also overestimated by UHF as well as ROHF for the same reasons that the g-shifts are exaggerated.

**Table 4:** Comparison of Calculated and Experimental EPR Parameters for [Cu­(NH_3_)_4_]^2+^ ^c^

	UHF	ROHF	UCCSD	SORCI^b^	exp.
Δ*g*_||_	416.2	476.7	261.8	243	241
Δ*g*_⊥_	89.1	93.7	57.2	55	47
*A*_*iso*_^*Cu*^	–616.9	–1.5	–551.3	–362	
*A*_*dip*;||_^*Cu*^	–644.6	–652.8	–551.8	–577	
*A*_*dip*;⊥_^*Cu*^	322.3	236.4	275.9	288	
*A*_*orb*;||_^*Cu*^	549.3	622.8	378.0	345	
*A*_*orb*;⊥_^*Cu*^	121.7	123.0	85.6	77	
*A*_*total*;||_^*Cu*^	–710.0	–649.0^a^	–725,1	–591	(−)586
*A*_*total*;⊥_^*Cu*^	–172.2	–257.5^a^	–189,8	3	∼(-?)68
*A*_*iso*_^*N*^	20.5	8.2	29.2	29	
*A*_*dip*;||_^*N*^	4.5	3.0	7.0	7.2	
*A*_*dip*;⊥_^*N*^	–2.3	–1.5	–3.5	–3.6	
*A*_*total*;||_^*Cu*^	25.0	11.1	36.1	36.4	39.1
*A*_*total*;⊥_^*Cu*^	18.2	6.6	25.6	25.3	31.7

a Using the UHF isotropic hyperfine because ROHF cannot represent spin-polarization.

b Reference 70.

c All results in this work were obtained with the X2C Hamiltonian and the X2C-SVPall basis set. S-functions were decontracted for hyperfine calculations. All hyperfine couplings are given in MHz and g-shifts in ppt

We note in passing the excellent agreement between the results of the more than twenty-year-old SORCI calculation and the UCCSD response results. With the exception of the Fermi contact contribution to the copper hyperfine coupling, both sets of calculations are also in very good agreement with experiment, which underlines the importance of dynamic electron correlation in transition-metal complexes. The isotropic copper hyperfine coupling is still problematic due to the complicated physics of the core-level spin-polarization.

It is evident that the results of these calculations do not lead to an enthusiastic endorsement of the ROHF method for the calculation of transition-metal EPR properties in such relatively simple coordination compounds. However, that was also not to be expected: the ROHF calculations fail to be accurate in all the expected ways. However, the results show that the methodology is working properly and, at least in this author’s opinion, are encouraging for building more accurate correlation approaches on top of the g-ROHF or the CSF-ROHF treatment.

### 4.5 g-Tensors of Antiferromagnetically Coupled Dimers: Manganese Dimer

As a more advanced example of the application of the g-ROHF (here, CSF-ROHF) response theory, a very typical antiferromagnetically coupled transition-metal complex was revisited. The molecule [Mn­(DTNE)­(μ-O)_2_(μ-OAc)]^2+^ (DTNE = 1,2-bis­(1,4,7-triazacyclonan-1-yl)­ethane; in the following simply referred to as MnDTNE) was studied in great detail using advanced paramagnetic resonance techniques alongside broken-symmetry DFT calculations in ref 79. Chemically speaking, the complex features antiferromagnetic coupling between a Mn­(III) (d^4^, S = 2) and a Mn­(IV) (d^3^, S = 3/2) ion to a total ground state spin of S_t_ = 1/2 with seven unpaired electrons.

The structure of the molecule was optimized using broken-symmetry DFT together with the X2C Hamiltonian, TPSSh functional, and the X2C-TZVPPall basis set. Scalar relativistic X2C calculations were then performed using the CSF-ROHF method for the high-spin state (S_t_ = 7/2) and the antiferromagnetic state and the respective g-tensors and manganese hyperfine couplings were calculated. For comparison, UHF calculations were carried out for the high-spin state (M_S_ = 7/2) and the broken-symmetry states with M_S_ = 1/2. We also performed a CASSCF­(7,7) calculation for the low-spin state for comparison with CSF-ROHF. All single-point calculations were done with the X2C-SVPall basis set and the matching auxiliary basis.

The geometric and electronic structures of MnDTNE are shown in Figure 4 and hold no surprises. The two metal sites are in a distorted octahedral coordination environment and exist locally in high-spin states with the d^4^ Mn­(III) site (left) having the configuration (t_2g_)^3^(e_g_)^1^ and the d^3^ Mn­(IV) site (right side) having a (t_2g_)^3^(e_g_)^0^ configuration. Here, t_2g_ and e_g_ are referring to the irreducible representations under which the metal d-based molecular orbitals transform in the parent O_h_ group.

**Figure 4:**
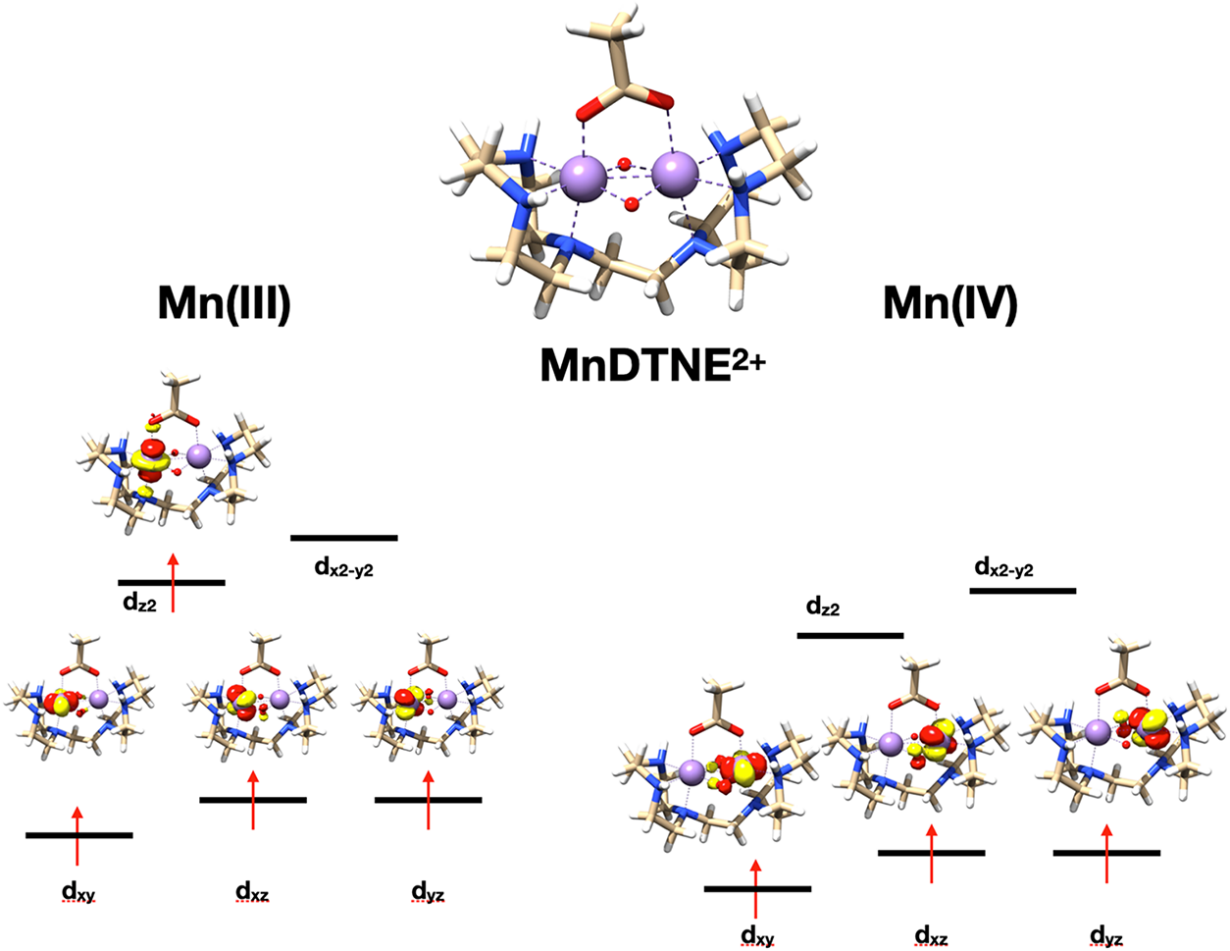
Geometric and electronic structure of MnDTNE^2+^ in its high-spin S = 7/2 state. Shown are the localized high-spin ROHF orbitals.

While this electronic structure description is in line with the basic principles of coordination chemistry, it is instructive to compare the optimized orbitals for the CSF-ROHF low-spin and the broken-symmetry UHF state. It was discussed in ref 80 and extensively used in the coordination chemistry community subsequently, that an illuminating way to visualize the electronic structure of such broken-symmetry solutions is based on the application of the corresponding orbital transformation.^81^ This transformation consists of separate unitary transformations of the spin-up and spin-down orbitals that bring them in maximum coincidence such that each occupied spin-up orbital overlaps with at most one spin-down orbital. If the orbital pairs are ordered according to spatial overlap, one first finds all orbitals that nominally belong to closed shells with overlaps very close to one, followed by the ‘magnetic pairs’ that have overlaps significantly smaller than one. These overlaps visualize the antiferromagnetic coupling ‘pathways’ that the system uses. Finally, the unmatched spin-up orbitals correspond to the majority of spin sites in such a calculation.

In Figure 5 the corresponding orbitals of a broken-symmetry UHF calculation on MnDTNE are compared to the singly occupied orbitals of the S = 1/2 g-ROHF calculation with the spin–coupling pattern + + + + – – – corresponding to the antiferromagnetic coupling of the four singly occupied orbitals on the Mn­(III) site and the three singly occupied orbitals on the Mn­(IV) site. It is apparent that the corresponding orbital pairs of the broken-symmetry solution have more extended tails that leap via the bridging ligands onto the other site, as evidenced by nonzero values of the spatial overlap integrals (Figure 5). This is possible because the spin-up and spin-down orbitals are already orthogonal through their spin parts and consequently, there is no constraint on the orthogonality of their spatial parts. It is exactly this delocalization and the corresponding nonorthogonality that provide the energetic lowering of the broken-symmetry solution corresponding to the antiferromagnetic coupling. Hence, the larger the corresponding orbital overlaps, the more pronounced the antiferromagnetic coupling will be. In the present case, the intuitive result is obtained that the strongest antiferromagnetic pathway proceeds through the bridging oxo-ligands (S = 0.073), whereas the π (S = 0.04) and δ (S = 0.02) pathways are weaker.

**Figure 5:**
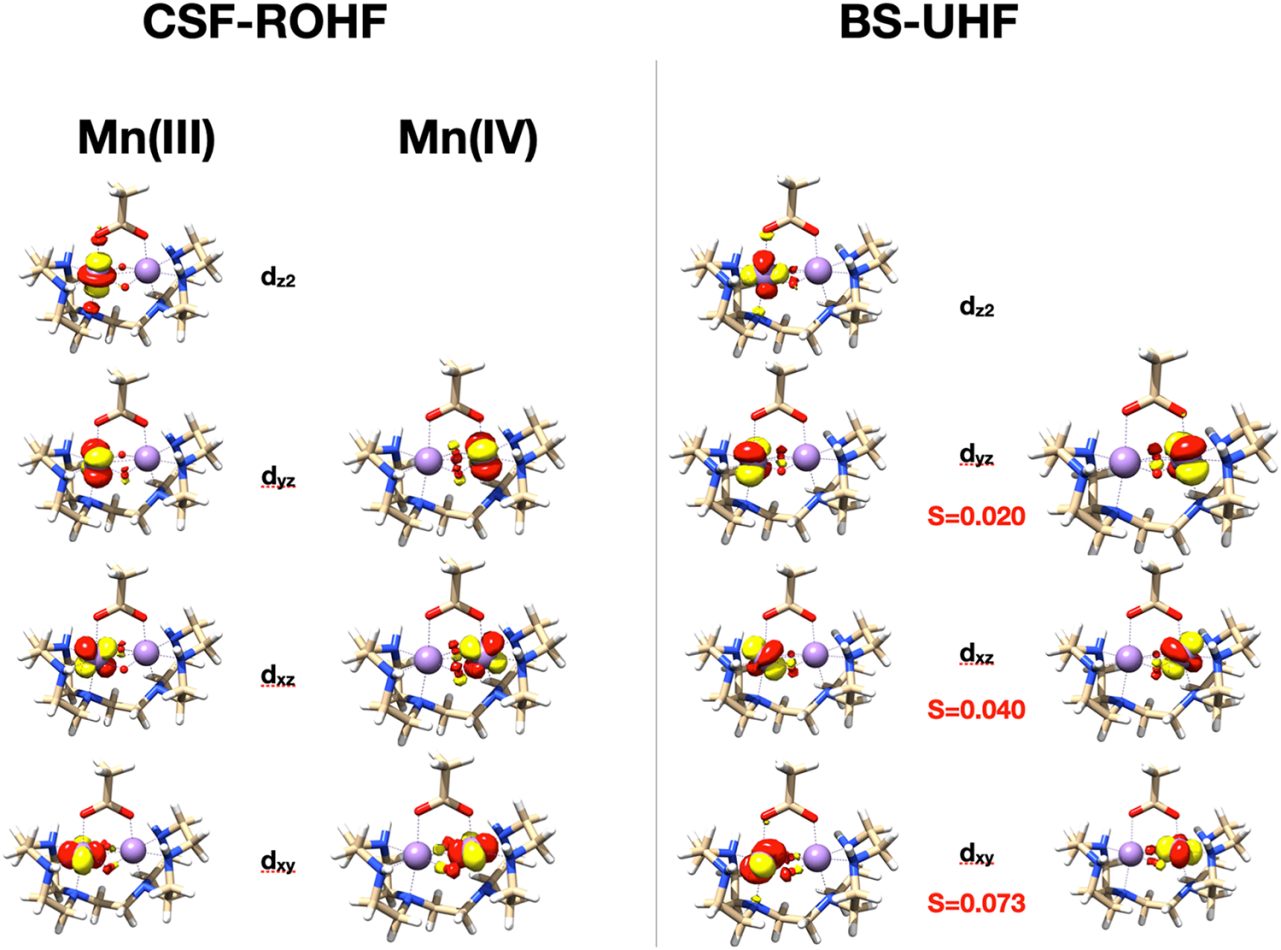
Comparison of the magnetic orbitals in the low-spin state of MnDTNE from CSF-ROHF (left) and broken-symmetry UHF (right) calculations. For the UHF solution, the corresponding orbital pairs are plotted together with their spatial overlap integrals. The CSF-ROHF orbitals are all rigorously orthogonal.

While the picture painted by the broken-symmetry UHF calculations is largely physically correct and the broken-symmetry electron density is physically sound, the corresponding spin density is not. In fact, the spin density contributions to the individual sites will be largely overestimated. The fact that the broken-symmetry spin density is fundamentally flawed is most easily understood with reference to the case where the antiferromagnetic coupling leads to an overall singlet state. While a true singlet state has zero spin density everywhere in space, the broken-symmetry solution will give a region with a large positive spin density and a region with a correspondingly large negative spin density such that only the integrated spin density is zero. We come back to this point below and in Figure 6.

**Figure 6:**
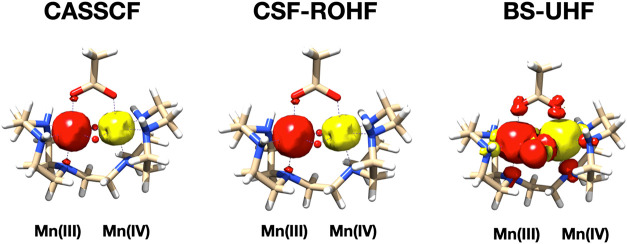
Comparison of the spin density distribution in MnDTNE in the low-spin state from CASSCF­(7,7) (left panel), CSF-ROHF (middle panel), and broken-symmetry UHF (right panel). All densities were contoured at 7/–0.003 Electrons/Bohr^3^. Red = positive, yellow = negative spin density.

The CSF-ROHF solution behaves fundamentally differently. Since in this orbital optimization all singly occupied orbitals are constrained to be orthonormal, the delocalization that stabilizes the broken-symmetry state is not possible. Thus, unsurprisingly, the CSF-ROHF optimized orbitals are virtually indistinguishable from the high-spin ROHF orbitals. Due to the lack of stabilization by delocalization, the CSF-ROHF antiferromagnetic state will always be higher in energy than the corresponding high-spin state. This is physically completely correct, since in this picture, the antiferromagnetic coupling will come in through the mixing of ionic configurations that correspond to metal-to-metal charge transfer (for a review, see^82^). Thus, while the CSF-ROHF method is an excellent starting point for a correlated ab initio treatment of the exchange coupling phenomenon, the method itself will only deliver ferromagnetic coupling.

The spin density delivered by the CSF-ROHF method is fundamentally physically correct and belongs to the spin-eigenfunction indicated by the chosen spin–coupling scheme. For the case at hand, the spin–coupling coefficients γ are ${\gamma \;}_{\mathrm{Mn}\left(\mathrm{III}\right)}=\frac{1}{2}$ for the Mn­(III) site and ${\gamma \;}_{\mathrm{Mn}\left(\mathrm{IV}\right)}=-\frac{1}{3}$ for the Mn­(IV) site. Thus, the four singly occupied orbitals on the Mn­(III) site will contribute 2 excess spin-up electrons to the integrated spin density, and the three singly occupied orbitals on the Mn­(IV) will contribute one excess spin-down electron, leading to a total spin density of 1 excess spin-up electron. On the other hand, in the broken-symmetry solution, the Mn­(III) sites will contribute 4 excess α electrons and the Mn­(III) site three excess spin-down electrons. This unphysically large overestimation of the spin density in the broken-symmetry case is clearly visible in the comparison spin density plot in Figure 6.

In order to illustrate this point further, we carried out CASSCF­(7,7) calculations for the low-spin state where the active space consists of the singly occupied orbitals found in the low-spin CSF-ROHF calculations. These calculations converge smoothly starting from the CSF-ROHF orbitals and lead to an energy merely 0.4 mEh or 86 cm^–1^ below the CSF-ROHF solution. This energy lowering is brought in by the ionic configurations that are included in the CASSCF treatment but not in the CSF-ROHF treatment. It is well-known that this mixing is grossly underestimated by the CASSCF method, and consequently, predictions of Heisenberg exchange coupling parameters are very poor at the level of a minimal CAS. Nevertheless, it is reassuring that the Löwdin spin-populations at the Mn­(III) and Mn­(IV) center are nearly identical and amount to 1.916 and 1.910 on the Mn­(III) site and −0.941 and −0.933 on the Mn­(IV) sites for CSF-ROHF and CASSCF, respectively. Thus, in the present case, the CSF-ROHF method is an excellent substitute for CASSCF at a much lower computational cost and essentially no limitations in “active space” size. This underlines that a thorough understanding of the electronic structure situation under investigation can lead to very large computational savings together with tools that are tailored to the problem at hand.

In ref 79, we had put forward a model that allowed us to estimate the g-tensor of the antiferromagnetic state from the calculated g-tensors of the high-spin and broken-symmetry states. It is customary to think about the system g-tensor of such a relatively weakly antiferromagnetically coupled dimer in terms of hypothetical “site” g-tensors ***g*** ^(1)^ and ***g*** ^(2)^. The system g-tensor is then obtained by

56 $$g=c_{1}g^{\left(1\right)}+c_{2}g^{\left(2\right)}$$

For the system at hand, *c*_1_ = 2 for the majority spin Mn­(III) site and *c*_2_ = −1 for the minority spin Mn­(IV) site. The model then states that these site g-tensors might be calculated from the high-spin and broken-symmetry states as

57 $$\begin{array}{l} g^{\mathrm{Mn}(\mathrm{III})}=\frac{1}{2S_{1}}\left(g^{\left(\mathrm{HS}\right)}M_{S}^{\left(\mathrm{HS}\right)}+g^{\left(\mathrm{BS}\right)}M_{S}^{\left(\mathrm{BS}\right)}\right)\\ g^{\mathrm{Mn}\left(\mathrm{IV}\right)}=\frac{1}{2S_{2}}\left(g^{\left(\mathrm{HS}\right)}M_{S}^{\left(\mathrm{HS}\right)}+g^{\left(\mathrm{BS}\right)}M_{S}^{\left(\mathrm{BS}\right)}\right) \end{array}$$

where *S*_1_ = 2, *S*_2_ = 3/2, *M*_*S*_^(*HS*)^ = 7/2 and *M*_*S*_^(*BS*)^ = 1/2. The ability to calculate the UHF high-spin and broken-symmetry solutions as well as the ROHF high-spin and the antiferromagnetic state S_t_ = 1/2 state, directly puts us in a position, for the first time, to evaluate the assumptions of the model.

Experimentally, the low-spin ground state of MnDTNE shows a nearly axial g-tensor with two *g*-values close to the free-electron value and the third value below *g*_*e*_ at 1.9838. Qualitatively, this is in excellent accord with the CSF-ROHF prediction of the three principal *g*-values of 1.9701, 2.0105, and 2.0118. Thus, the symmetry of the g-tensor is correctly predicted by the fact that the one larger g-shift is below *g*_*e*_ and the other two are close to *g*_*e*_. The absolute value of the negative shift is, unsurprisingly, somewhat overestimated. If one uses the calculated high-spin *g*-values (1.9889, 1.9925, 1.9967) and broken-symmetry *g*-values (1.9587, 1.9989, 2.0514) together with eqs 56 and 57 and takes into account that the two g-tensors do not diagonalize in the same coordinate system, one obtains 1.9279, 1.9857, and 2.0820 for the low-spin state, a result that is clearly inferior to the more rigorous direct CSF-ROHF calculation. The broken-symmetry B3LYP-based results in ref 79, where somewhat better than the UHF results obtained here, indicating that part of the problem is the rather low quality of the UHF method for open-shell transition-metal complexes.

Taken together, the results of this section indicate that the CSF-ROHF (or, more generally, the g-ROHF) method provides qualitatively correct results for antiferromagnetically coupled systems and provides an elegant and efficient pathway to electric and magnetic response properties. The principal shortcoming of the method is the modest accuracy of the underlying Hartree–Fock method. This subject will be addressed in forthcoming publications.

### 4.6 Complex Spin Couplings in Metal-Radical Assemblies: Iron-Complex

As a representative member of the class of metal–ligand radical systems, we have chosen the complex [Fe­(GMA)­(pyridine)]^+^, where GMA stands for glyoxal-bis­(2-mercaptoanil). The complex was studied in great detail using Mössbauer and EPR spectroscopy coupled to broken-symmetry DFT calculations in ref 83. This system is particularly fascinating because in the original analysis, it was concluded that there is a hitherto unknown bonding situation present in which the GMA ligand coordinates in its first excited triplet state to an intermediate spin Fe­(III) ion to give a resulting total spin of S_t_ = 1/2. This phenomenon was given the name “excited-state coordination” and it led to the formulation of a “metal-field theory”,^84^ complementary to the familiar ligand-field theory that is the cultural basis for much of coordination chemistry. The strong antiferromagnetic coupling between the metal ion and the ligand was identified as the driving force for the excited-state coordination.

The structure of the complex was optimized in the same way as MnDTNE using broken-symmetry DFT together with the X2C Hamiltonian, TPSSh functional, and the X2C-TZVPPall basis set. Calculations were then performed using the CSF-ROHF method for the high-spin state (S_t_ = 5/2) and the antiferromagnetic (S_t_ = 1/2) state, and the respective g-tensors were calculated. As for MnDTNE, these calculations were performed with the X2C scalar relativistic Hamiltonian, the X2C-SVPall basis set, and the matching auxiliary basis set.

The results collected in Figure 7 nicely show the electronic structure that was postulated in ref 83 for the ground state of FeGMA. One finds three unpaired electrons on the intermediate spin Fe­(III) center antiferromagnetically coupled to the first excited triplet state of the GMA ligand that is formed by promoting one electron from the ligand HOMO (a thiolate-based lone pair) to the ligand LUMO (the π* LUMO of the diimine motif).

**Figure 7:**
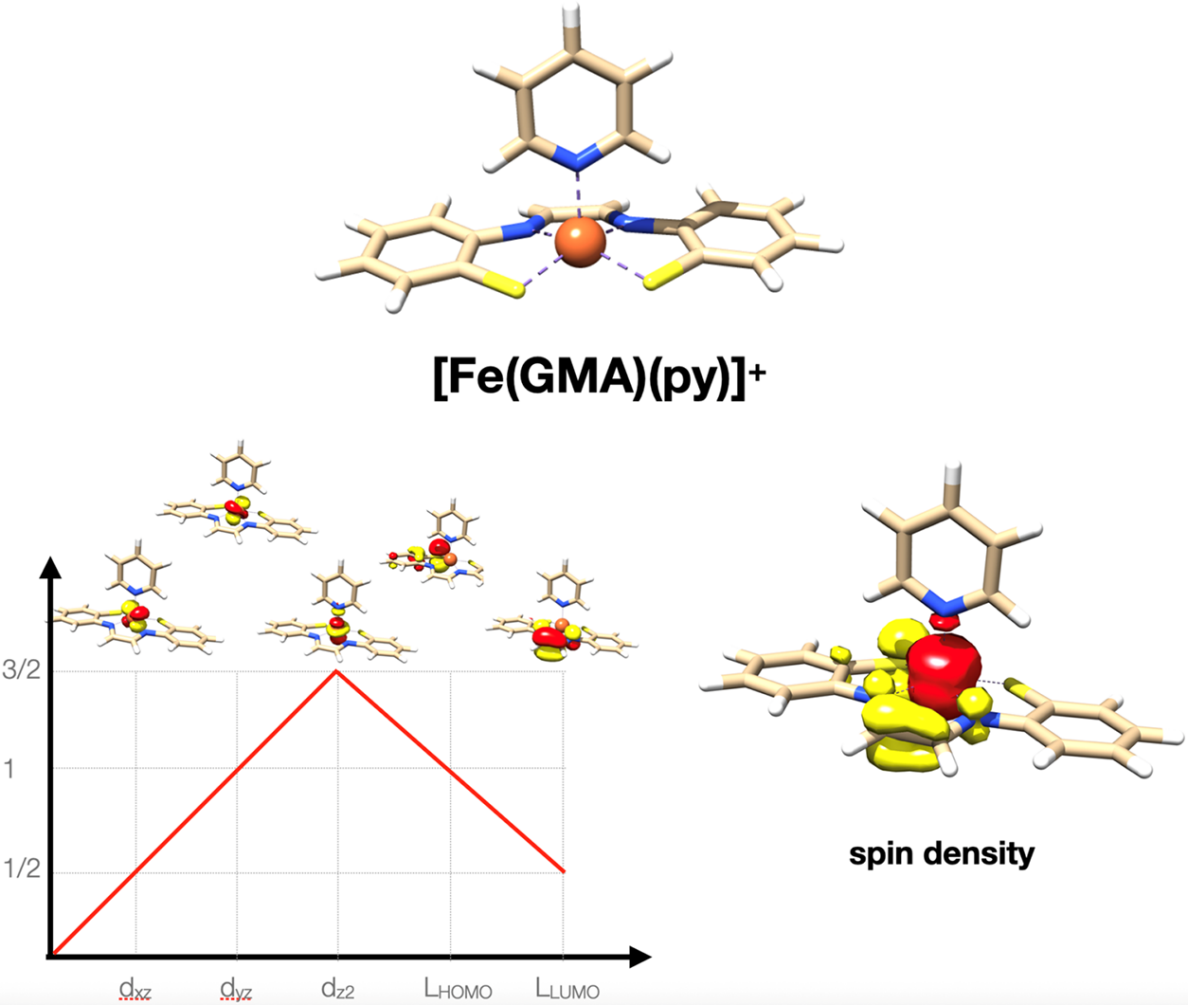
Geometric and electronic structure as well as spin density in the S_t_ = 1/2 ground state of [Fe­(GMA)­(py)]^+^ from CSF-ROHF calculations.

The calculated g-tensor of the S_t_ = 1/2 ground state shows the two g-values, all of the free-electron g-value, and one value close to it (*g* = 2.0007, 2.0393, 2.0737). The analysis in ref 83 concluded that these reflect the intrinsic g-value of the intermediate spin Fe­(III) site, enhanced by 5/3 due to the spin coupling. Experimentally, no accurate g-tensors were reported, but it was concluded that the EPR is consistent with spectra obtained for related intermediate spin Fe­(III) systems that feature positive g-shifts with g-values between 2.0 and 2.2, consistent with the results of the CSF-ROHF calculations. We note in passing that the energy of the low-spin state is calculated to be some 16 kcal/mol below the high-spin state and about 8 kcal/mol below the “pure” doublet state featuring a low-spin Fe­(III) center and a closed-shell ligand. However, a low-spin Fe­(III) center would feature first-order SOC, leading to much larger g-shifts than the one observed. Since the ground state spin state is definitely S_t_ = 1/2, the calculated “excited-state coordination” metal-radical state still offers the best explanation for the experimental findings.

## 5 Discussion

In this paper, the response of a general restricted open-shell Hartree–Fock (g-ROHF) wave function was derived and implemented. While there certainly have been response formulations for the ROHF high-spin case, the author is not aware of another implementation of the g-ROHF response. This development is considered to be an important contribution to the quantum chemistry of open-shell systems, which opens the door for many future applications and extensions.

The present development enhances the applicability of the general ROHF scheme considerably by giving access to electric and magnetic response properties that do not suffer from spin contamination. That holds even for cases with nontrivial coupling between open-shell electrons, for example, in antiferromagnetically coupled transition-metal complexes, in metal-radical assemblies, or in the excited states of closed- and open-shell molecules. The present approach allows one to specifically target a desired spin–coupling state and obtain the properties of such a state. The method is computationally efficient and will not cost more than, for example, a UHF calculation on the same system. The generality of the treatment should not be underestimated as it will be one of the cheapest and most convenient methods to calculate excited-state response properties from properly spin-adapted wave functions that is currently available. It is possible to use an existing CASSCF response infrastructure in order to simulate the behavior of g-ROHF because, for the most part, all that is required is to replace the CASSCF Fock and reduced density matrices with their g-ROHF counterparts in order to obtain equivalent response results. In fact, for the high-spin case, CASSCF and g-ROHF are identical. A referee to this paper has informed the author that they had accomplished this previously, but not published the results. Clearly, this is a valid approach to obtain an implementation of the g-ROHF methodology without too much development effort, provided that the much more complex CASSCF response is already available. However, computationally, the CASSCF-based approach will be much more demanding with respect to the increase in system and active space sizes.

Many of the properties of the g-ROHF approach are also shared by spin-flip approaches.^28–31,85–89^ The main differences may be viewed as the contrast between a bottom–up (g-ROHF) approach and a top–down (spin-flip) approach. In g-ROHF, the wave function, energy, and responses are all constructed in detail from the bottom up with explicit recourse to the nature of the underlying wave function. In spin-flip approaches, one starts from a wave function that has all open-shell electrons aligned and then applies spin-flip operators in order to create the desired CSFs and their properties. The relative merits of both approaches warrant further study once the g-ROHF method has undergone further development.

In terms of the g-ROHF method, one is clearly still bound by the limited accuracy of the underlying Hartree–Fock model. Hence, despite being qualitatively correct in the more intricate electronic situations mentioned, one cannot really expect high accuracy. However, it can be argued that the g-ROHF method is an excellent starting point for more accurate electronic structure methods. The recent development of the general-spin ROCIS method^55,56^ is a good example for such a post g-ROHF treatment. Ultimately, combining g-ROHF with coupled-cluster expansion is a very worthy and ambitious research goal that we are actively pursuing. In addition, the generalization of the method to density functional theory appears to be readily achievable and will be pursued in the future.

A referee has pointed out that the fact that the g-ROHF method always delivers ferromagnetic coupling is contrary to the claim of it being “qualitatively correct”. While this is certainly a valid criticism, in this author’s opinion, the treatment is still qualitatively correct, since the antiferromagnetically coupled CSF is still the dominant contribution in the wave function while the shortcomings of not properly including the ionic components required for numerical accuracy are well understood.

In addition to the properties that were already discussed in this paper, obvious extensions include the implementation of nuclear perturbations and gauge including atomic-orbital (GIAO) magnetic field-like perturbations. Their implementation is merely a technicality and will be accomplished in the near future. This will open up the door for the implementation of analytic second derivatives and related properties such as the diagonal Born–Oppenheimer correction or vibrational circular dichroism for complex open-shell molecules.

An exciting prospect is also the calculation of nuclear Hessians and other properties for electronically excited singlet states at virtually the same computational cost as a closed-shell calculation. Furthermore, the method lends itself very well to the calculation of excitation energies and optical spectra in the framework of a random-phase scheme, as will be explored in a forthcoming paper.

Last but not least, it was shown in this paper that the g-ROHF approach offers a very convenient route to the separate calculation of first- and second-order contributions to magnetic properties in a controlled manner. Since intrashell rotations are excluded from the response treatment, any first-order contributions to, say, the electronic g-tensor are missing, and only the second-order corrections arising from the remainder of the electronic system are captured in the response approach. Thus, the intrashell, first-order contributions for, say, a degenerate doublet can be calculated with a very small CI expansion while the response treatment takes care of the rest. This addresses, if not solves, an old problem of quasi-degenerate perturbation theory of magnetic properties, namely, the fact that the QDPT treatment is truncated and does not resolve the contributions from the electronic structure responses that arise outside the active space. We are looking forward to exploring this avenue further.
